# Challenges to the adoption of climate-smart agricultural practices among smallholder farmers in semi-arid Ghana

**DOI:** 10.1371/journal.pone.0355679

**Published:** 2026-08-13

**Authors:** Juliana Toboyee, Mildred Naamwintome Molle, Kenneth Peprah, Raymond Aabeyir, Sulemana Ansumah Saaka, Isaac Luginaah

**Affiliations:** 1 Department of Environment and Resource Studies, University of Business and Integrated Development Studies Wa, Upper West Region, Ghana; 2 Department of Geography and Environment, Western University, London, Ontario, Canada; 3 Department of Geography, University of Business and Integrated Development Studies Wa, Upper West Region, Ghana; University of Nevada Las Vegas, UNITED STATES OF AMERICA

## Abstract

With the increasing recognition of climate change threats to agricultural productivity and food security, it is crucial to explore farming practices that increase productivity, adapt to the effects of global climate change, while lowering greenhouse gas emissions. This study investigates the factors that influence the adoption of climate-smart agricultural (CSA) practices among smallholder farmers in semi-arid Ghana, specifically, the Upper West Region (UWR) of Ghana. The study surveyed 400 smallholder farmers from eleven (11) Communities and employed stratified random sampling, and a logistic regression model for data analysis. The results revealed that knowledge of CSA benefits or motivation factors such as improved crop yields (OR=10.356; p < 0. 001), reduced input cost (OR=4.855; p < 0.05), influenced the decision of smallholder farmers to adopt CSA practices. Also, availability of support systems such as access to agricultural extension services (OR=10.358; p < 0.001) and CSA training (OR=4.374; p < 0.05) were significantly associated with the adoption of CSA practices. The findings also shows that demographic factors shape the adoption of CSA practices. Farmers within the ages of 26–45 were less likely to adopt CSA practices (OR = 0.050; p < 0. 05), while farmers with formal education were more likely to adopt CSA practices (OR = 9.262; p < 0.001) after controlling for support system variables. Based on the findings, improved crop yields, resilience, reduced input, support from Agricultural Extension Agents (AEAs), financial support, training, and government policies are factors that influence CSA adoption among smallholder farmers in the study area. The study calls for policymakers and non-governmental organizations (NGOs) to enhance financial support systems and extension services to farmers, as well as provide more training on CSA. Government policies should also be focused on education and sensitization to upscale the adoption of CSA practices for its maximum benefits.

## 1. Introduction

Climate change is a significant global threat to development, with far-reaching implications for the agricultural sector. It affects the biophysical and socioeconomic elements that influence temperature, precipitation, CO_2_ concentration, pests and diseases, agricultural productivity, and market prices [1]. Although climate change has both positive and negative effects on crop yields and livestock production, depending on crop or livestock type, location, and level of adaptation capacity, the overall trend of its effects is expected to be unfavourable, particularly in low-latitude regions [2].

Various factors, including changing precipitation patterns, extreme weather events, and rising temperatures, cause crop yield losses [1,3]. Due to erratic weather patterns, crop failures, and declining incomes, these climate-related risks present serious obstacles to rural livelihoods [3]. Ultimately, declining crop yields threaten global food security, underscoring the pressing need for adaptation strategies to ensure a stable food supply [1]. Reduced agricultural productivity has a particularly negative economic impact on developing nations where agriculture is a significant source of income. Uncertainty in agricultural production is exacerbated by the trend toward more frequent extreme weather events, which particularly affect staple crops such as maize, rice, and soybeans. Global yields could drop by 12–20% by the end of the century if innovative adaptation measures are not taken [1]. Rising temperatures also cause thermal stress in livestock, reducing productivity by decreasing feed intake, slowing growth rates, and impairing reproductive outcomes [4].

In Ghana, agriculture is a critical sector of the economy, employing 45.38% of the total workforce and contributing to food security and poverty alleviation. However, it is one of the sectors adversely affected by climate change, as most farmers rely on rain-fed agriculture [5,6]. According to [7], climate change has altered rainfall patterns and farming seasons in Ghana, making it difficult for farmers to predict rainfall onset and cessation and to organize their agricultural activities. This has led to reduced agricultural yields, increased pest and disease outbreaks, and food insecurity among farmers [8]. Furthermore, climate change has affected farmers’ local cultures and indigenous knowledge of farming, requiring them to adapt to new techniques and technologies that may conflict with their traditional values and beliefs [7].

The detrimental impacts of climate change on agriculture have prompted the emergence of programmes that support sustainable practices, such as Climate-Smart Agriculture (CSA) [9]. These programmes highlight the importance of farmers implementing strategies to address climate change, boost agricultural productivity, and ensure food security. CSA is widely recognized as a viable approach to addressing the multifaceted challenges of climate change and food insecurity in Ghana, Africa, and beyond.

CSA is a strategy for increasing the resilience and productivity of agricultural systems while lowering greenhouse gas emissions and achieving socioeconomic benefits [10]. CSA supports smallholder farmers in enhancing their incomes, food security, and market access.

The introduction of CSA practices in Ghana, such as minimum tillage, intercropping, soil nutrient amendment, and drought-tolerant crops, has been shown to increase soil carbon stocks, reduce water and fertilizer use, and improve crop productivity.

Belay et al. [11] investigated the effects of CSA practices on smallholder farmers in southern Ethiopia, an area susceptible to climate change. According to the research, smallholder farmers who implemented CSA practices, such as crop management, water conservation, and agroforestry, experienced a 20.30% increase in average annual per-hectare farm income compared with those who did not. Furthermore, by increasing crop yields and diversifying production, CSA practices directly affect food security [11]. Also, it was reported that those who adopted CSA practices had better access to nutrient-dense foods, as evidenced by significantly higher food consumption scores (ranging from 6.27 to 8.15) among adopters compared with non-adopters. Additionally, a crucial element affected by the adoption of CSA practices is market access. Through CSA, smallholder farmers can participate in value chains, reach markets, and sell their produce, thereby supporting market-oriented agriculture. Transferring CSA knowledge, connecting farmers with markets, and offering technical assistance depend on efficient extension services. The benefits of CSA are well documented in the literature; however, factors influencing the adoption of CSA practices among smallholder farmers in semi-arid environments are not exhaustive, which still undermines the potential of adopting CSA practices among these farmers in a fragile area. For instance, agricultural extension, input costs, training on CSA practices, government policies, and financial incentives are critical factors in the adoption of CSA among poor and vulnerable smallholder farmers. Understanding these factors and their nature will support policymakers and farmers in decision-making regarding CSA adoption. The study examines factors influencing CSA adoption practices among smallholder farmers in semi-arid Ghana, specifically the Upper West Region.

## 2. Theoretical context

This study draws theoretical insights from the theory of planned behavior [12]. The theory of planned behavior is commonly used in health studies to understand factors that influence individuals’ decisions to undertake a particular activity, such as self-breast examination and tobacco/cigarette intake [13,14]. The useful application of this theory within the context of climate-smart agriculture will facilitate the understanding of factors influencing farmers’ decisions to adopt CSA practices on their farms. Our study drew on three constructs of the theory- attitudes, subjective norms, and perceived behavioral control [15–17].

Attitude is the degree to which a person views a certain behavior favorably or negatively. It is an individual’s overall evaluation or thoughts about doing something. An attitude towards positive behavior increases the possibility of intention to perform it [17]. Behavioral beliefs and outcome judgments make up attitude. It can also be divided into affective and instrumental attitudes. The person believes the action is delightful or unpleasant with an affective attitude, and the behavior is beneficial or detrimental with an instrumental attitude. In the context of CSA adoption, farmers may weigh the benefits, which include enhancement of agricultural productivity, improved food security, increased farm resilience against climate change, and reduced input cost [18–21]. Subjective norm, the second construct is a social pressure to perform or not to perform a given behaviour [18,22]. The subjective norm is the support given by others to undertake a particular activity. Subjective norms can be injunctive (whether others encourage you to do the behaviour.) or descriptive (whether others in a person’s social group engage in the behaviour) [23]. In the context of CSA, farmers may consider whether other farmers, family members, or extension officers support or discourage adopting CSA practices. Farmers often look to their peers for guidance and validation. If they observe that other farmers in their community are successfully adopting climate-smart practices and receiving positive feedback or benefits (such as increased yields or improved soil health), they may be more inclined to follow suit. This peer influence can create an effect where the adoption of CSA becomes a common practice within the community. Additionally, subjective norms play a crucial role in disseminating information about CSA. If extension officers highlight the significance of CSA, farmers are more likely to embrace information, training, and resources that support their adoption of these practices [24].

The third construct perceived behavioral control [18], is important in the theory since it corresponds to people’s perceptions of the ease or difficulty in conducting the activity of interest. Perceived behavioral control explains the capability and confidence an individual has in performing a particular behaviour [18]. Individuals who believe they lack the resources or opportunities to perform a certain behavior are unlikely to have high intentions to do so [25]. In the context of adopting CSA, perceived behaviour control can significantly influence farmers’ intentions and actual adoption in several ways, such as access to resources. If farmers perceive that they have access to the necessary resources to implement CSA, their perceived behaviour control will be higher, leading to a greater likelihood of adoption. Conversely, if they perceive a lack of resources, their confidence may diminish, reducing the likelihood of adopting CSA. If farmers also have a support system, such as extension services and training programs, this can enhance farmers’ perceived behavioral control. When farmers know they can seek help or guidance, they are more likely to feel capable of adopting CSA practices. The theory of planned behavior offers a structured framework for comprehending the adoption of CSA. It can be used to investigate the factors influencing the adoption of CSA practices among smallholder farmers in semi-arid Ghana.

### 2.1. Study context

**Fig 1:**
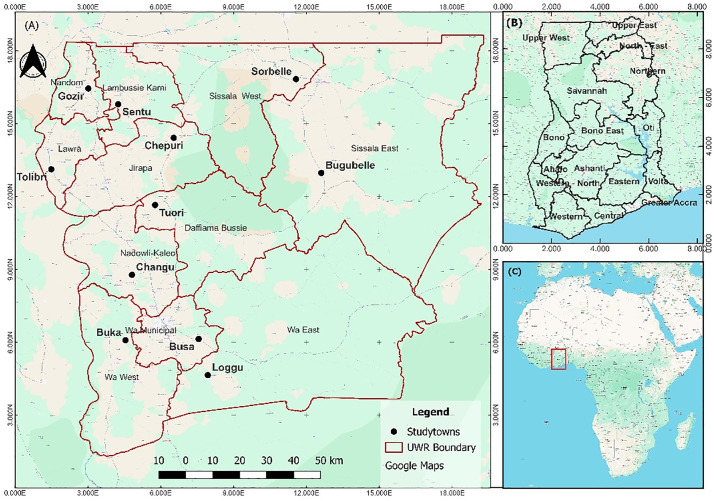
Map of the study area (prepared using ArGISPro Desktop).

The research was conducted in the Upper West Region (UWR) of Ghana. The UWR is a vast semi-arid area of 18,476 square kilometers located between longitudes 1° 36′ to 3° West and latitudes 9° 48′ to 11° North. Fig 1. shows a map of the UWR. The UWR is marked by perineal bushfire, partly due to the culturally embedded practice of using fire for various overlapping livelihoods within the same area. This is key to the annual occurrence of wildfires and the associated damage to crops, which create both direct and indirect links to food insecurity for smallholder families [26]. The region features drought-resistant scattered trees, including *Eucalyptus sp.* (eucalyptus), *Faidherbia albida* (Acacia albida), *Mangifera indica* (mango), *Adansonia digitata* (baobab), *Blighia sapida* (ackee), *Parkia biglobosa* (dawadawa), *Anacardium occidentale* (cashew), and *Vitellaria paradoxa* (shea).

The UWR is part of Ghana’s northern savannah ecological zone, where rainfed agriculture is the primary source of income and livelihood [27]. This region is home to 901,502 residents, and its savannah landscape experiences a single rainy season and a lengthy dry season known as the harmattan, typical of West Africa. Daily temperatures in the area fluctuate between 21 °C and 40 °C, while annual rainfall ranges from 840 mm to 1,400 mm [27]. These climatic factors result in alterations to the cropping calendar for smallholder farmers, ultimately affecting agricultural productivity since most farming activities depend on rainfall.

Agriculture in the UWR is mostly subsistence-based, with smallholder farmers engaging in crop production and livestock rearing. However, the dependence on traditional farming methods and limited access to modern agricultural technology and irrigation facilities increase the vulnerability of farming communities [27]. The region is susceptible to extreme climate stressors that significantly impact agricultural productivity, poverty, and food insecurity, water insecurity, and out-migration [28], and environmental pollution [29]. The socioeconomic difficulties and limited access to credit and agricultural inputs further reduce the farmers’ ability to adjust to changing climatic conditions [24]. The farmer-to-agricultural extension agent (AEA) ratio is relatively low, with one AEA to more than 5000 farmers in the UWR [27]. Establishing direct one-on-one contact between AEA and farmers is difficult due to limited resources. These and many more render the UWR particularly vulnerable to climate change impacts. In this context, embracing CSA practices is essential for creating a supportive environment that promotes sustainable agriculture and helps endure climatic disturbances.

The UWR’s culture is diverse, with various ethnic groups such as the Dagaaba, Waala, Sissala, and Brifo shaping the sociocultural dynamics in adopting new technologies, innovations, and agricultural practices. The study’s significance lies in the UWR’s reliance on agriculture and vulnerability to climate change. Also, this paper aligns with Inclusive Growth and Sustainable Development, key goals of Agenda 2063, which aims to eradicate poverty, achieve economic diversification, and ensure environmental sustainability.

It also aligns with Sustainable Development Goal 2, which aims to end hunger, improve nutrition, and promote sustainable agriculture, and Goal 13, which seeks to take urgent action to combat climate change and its impacts.

## 3. Method

### 3.1. Data collection and sample

A cross-sectional design was utilized for the study. Stratified random sampling was used to select 400 smallholder farmers from selected communities across the region in the UWR of Ghana, where CSA is promoted and practiced. All the study communities (Busa, Buka, Bugbelle, Loggu, Tuori, Chaangu, Chapuri, Tolibr, Sentu, Gozir, and Silbelle) had received some form of intervention from organizations that promote the practice of CSA, and this was one of the criteria for selecting them. The survey instrument was designed in the Kobo Collect Application and was pre-tested for validity and reliability before administration. Additionally, only respondents aged 18 years or older were included in the study. The survey collected information on demographic, ecological, environmental, and institutional factors relevant to CSA adoption. A letter of consent was read aloud to respondents, who were asked to either thumbprint or sign to consent to the commencement of the study. This study complied with the Declaration of Helsinki, ensuring that we obtained informed consent and that participants were aware of the study’s benefits and risks. Participants’ recruitment started from 7^th^ February 2025 to 19^th^ February 2025, and data collection was done between February 24^th^ and April 10^th^ across all the communities selected for the study. The Simon Diedong Dombo University of Business and Integrated Development Studies (UBIDS) Research Ethics Review Board granted ethical approval for the study (UBIDS/RERB/002/2025/02/04).

### 3.2. Measures

The dependent variable for this study is the adoption of CSA. In the survey, respondents were asked if they practice CSA. Responses to this question were coded as 1 = Yes and 0 = No. Guided by both the theoretical framework of the study and existing literature, contextually important covariates were included in the analyses of the factors influencing adoption of CSA practices among smallholder farmers in the UWR of Ghana. These variables included *demographics* such as age, gender marital status, and educational attainment; *CSA benefits or motivational factors* such as improvement of crop yields, farm resilience, and reduction of input cost; as well as *support systems* such as adequate support from extension officers, sufficient financial initiatives, education or training on CSA practices, government policies, and access to subsidies for the implementation of CSA. These variables were coded as follows: Age (18–25, 26–35, 36–45, 46–59 and 60+), marital status (0 = Never Married, 1 = Married, 2 = Widowed/Divorced), education (0 = No formal education, 1 = Formal Education), Improvement of Crop yields (1 = Yes, 0 = No), Farm resilience (1 = Yes, 0 = No), and reduction of input cost (1 = Yes, 0 = No), adequate support from extension officers (1 = Yes, 0 = No), sufficient financial initiatives (1 = Yes, 0 = No), education or training in CSA practices (1 = Yes, 0 = No), government policies (1 = Yes, 0 = No), and access to subsidies for the implementation of CSA (1 = Yes, 0 = No).

### 3.3. Analytical approach

Descriptive statistics, bivariate, and multivariable analyses were conducted in this study. A descriptive statistic is used to provide an overview of the study population. Also, bivariate analyses were conducted to understand the relationship between each predictor variable and the outcome variable CSA. Finally, multivariable logistic regression models were employed for multivariable analyses, to explore the collective relationship between the predictors and the outcome variables (i.e., CSA). Results from the regression models are reported in Odds Ratios (OR). Where (OR>1), farmers are more likely to adopt CSA practices, and with (OR<1, farmers are less likely to adopt CSA practices. (OR=1) indicates that CSA adoption is equally likely to occur in both situations. All statistical analyses were performed using STATA 18.0 software.

## 4. Results

### 4.1. Demographic characteristics of study participants

**Fig 2:**
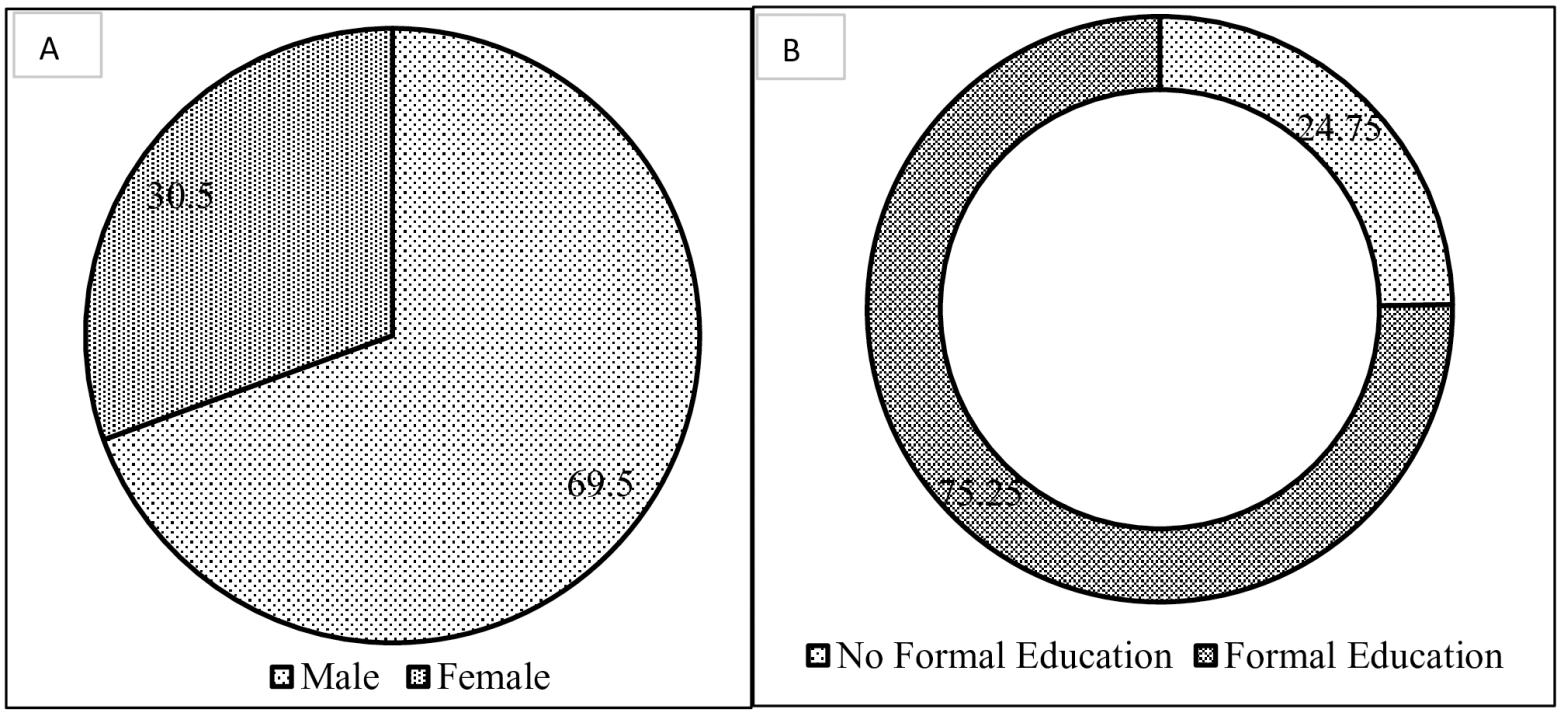
Sex distribution (A) and educational status (B) of respondents.

**Fig 3:**
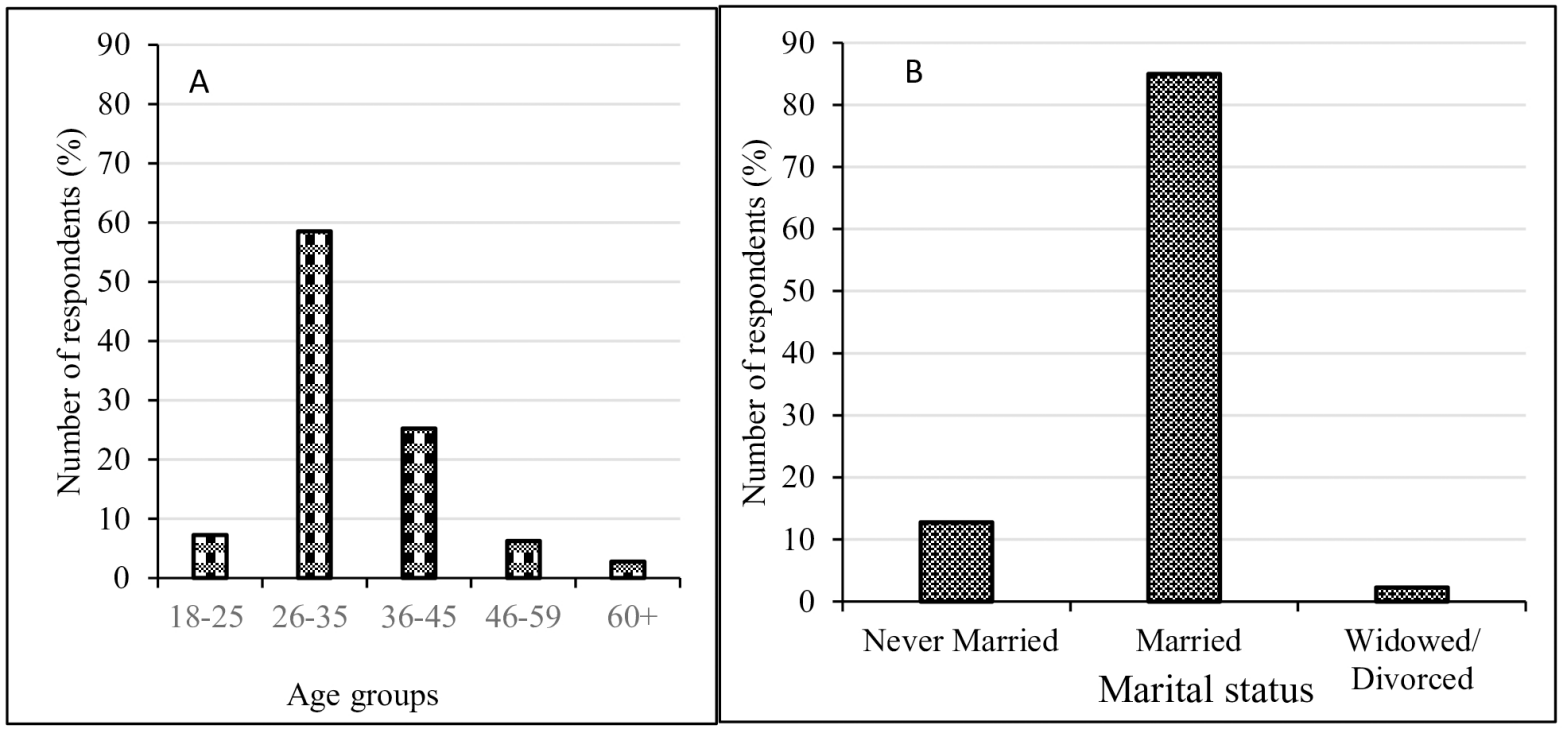
Age distribution and marital status of respondents.

The respondents’ demographic characteristics 69.50% were male and 30.50% were female (Fig 2A). In terms of education, 75.25% of respondents had some formal education, ranging from basic to tertiary levels (Fig 2B). The dominant age group among the respondents was 26–35 (58.5%) (Fig 3A), comprising a youthful and productive age group, with most of them (85%) being married farmers (Fig 3B). The skewness of respondents’ ages toward the 26–35 age group could be attributed to favourable government policies, such as the Planting and Rearing for Food and Jobs policy, which attracted many young people to farming. In the case of marital status, the skewness is attributed to farming being the primary source of livelihood for married families in rural Ghana, where the study was conducted.

Approximately 92.75% of respondents reported having adopted CSA practices on their farms (Table 1). Regarding the benefits of CSA, 92.25% of respondents indicated that the practice improves crop yields, and 60% indicated that it improves the climate change resilience of their farm crops. Also, 85% of respondents indicated that CSA reduced input costs for their farms. Regarding support for CSA implementation, 52% of respondents reported receiving support from extension agents for CSA practices. Moreover, 86% of respondents received some form of training or education on CSA practices. Only 40.25% of respondents reported that government policies encourage the implementation of CSA practices on their farms. Likewise, only 35.75% indicated that sufficient financial support exists for CSA implementation, and only 33.25% reported access to subsidies for its implementation.

**Table 1:** Description of variables and their measures.

Category of variable	variables	Measures and coding
Demographic	• Age • Gender • Education status • Marital status	• 18–25, 26–35, 36–45, 46–59, and 60+ • Female = 0, Male = 1, • No formal education = 0, Formal = 1 Education • Never Married = 0, Married = 1, Widowed/Divorced = 2
Motivational	• Improvement of crop yields • Farm resilience • Reduction of input costs	• Improvement of Crop yields (Yes = 1, No = 0) • Farm resilience (Yes = 1, No = 0) • Reduction of input cost (Yes = 1, No = 0),
Support systems	• Adequate support from extension officers • Sufficient financial initiatives • Education or training on CSA practices • Government policies • Access to subsidies for CSA implementation	• Adequate support from extension officers (Yes = 1, No = 0), • Sufficient financial initiatives (Yes = 1, No = 0), • Education or training in CSA practices (Yes = 1, No = 0), • Government policies (1Yes = 1, No = 0), • Access to subsidies for the implementation of CSA (Yes = 1, No = 0).

### 4.2. Bivariate analyses of factors influencing the adoption of CSA

Table 2 presents the results for bivariate analyses. At the bivariate level, smallholder farmers with formal education (OR=9.86; p < 0. 001) were significantly more likely to adopt CSA practices than those without formal educational attainment. Also, those who were married (OR=4.363; p < 0. 001) were significantly more likely to adopt CSA. Furthermore, benefits of CSA or motivational factors such as improved crop yields (OR=29.210; p < 0. 001) and reduced input costs (OR=15.293; p < 0. 001) were significantly more associated with the adoption of CSA in the study context. Respondents who received adequate support from agricultural extension officers (OR=3.716; p < 0. 05) were also more likely to adopt CSA practices than those who did not receive such support. Moreover, farmers who received training or education on CSA practices (OR=10.184; p < 0. 001) were more likely to adopt CSA practices on their farms.

**Table 2:** Descriptive statistics of study participants.

Variable	Frequency (%)
**Adoption of CSA**	
Agree	371(92.75)
Disagree	29 (7.25)
**Age**	
18-25	29 (7.25)
26-35	234 (58.50)
36-45	101 (25.25)
46-59	25 (6.25)
60+	11 (2.75)
**Gender**	
Male	278 (69.50)
Female	122 (30.50)
**Education**	
No Formal Education	99 (24.75)
Formal Education	301 (75.25)
**Marital Status**	
Never Married	51(12.75)
Married	340 (85.00)
Widowed/Divorced	9 (2.25)
**My Practice of CSA has improved my crop yields**	
No	31 (7.75)
Yes	369 (92.25)
**My Practice of CSA has improved the resilience of my farm**	
No	160 (40.00)
Yes	240 (60.00)
**My Practice of CSA has reduced input costs**	
No	60 (15.00)
Yes	340 (85.00)
**I receive adequate support from extension officers**	
No	192 (48.00)
Yes	208 (52.00)
**There are sufficient financial initiatives**	
No	257 (52.00)
Yes	143 (35.75)
**I have received education or training in CSA practices**	
No	56 (14.00)
Yes	344 (86.00)
**Government Policies**	
No	239 (59.75)
Yes	161 (40.25)
**I have access to subsidies for the implementation of CSA**	
No	267 (66.75)
Yes	133 (33.25)

### 4.3. Multivariate analyses of factors influencing the adoption of CSA

The multivariate logistic regression results of the study are presented in a nested model format in Table 3. We controlled for demographic variables in Model 1, the benefits of CSA in Model 2, and the support available for the practices of CSA in Model 3. With the demographic covariates in Model 1, farmers with formal education (OR=15.785; p < 0. 001) were more likely to adopt CSA practices on their farms compared to farmers with no formal education. Farmers who were married (OR=6.508; p < 0. 001) were six times more likely to adopt CSA on their farms for greater farm output compared to those who are never married. Consistent with results in Model 1, in model 2, farmers with formal education (OR=6.814; p < 0.05) as well as those who were married (OR=4.476; p < 0.05) were six time and four times more likely to adopt CSA practices, respectively. However, while no significant association was found between age and CSA in Model 1, in Model 2, farmers between the ages of 26–35 (OR=0.102; p < 0.1) were less likely to adopt CSA practices. Also in Model 2, benefits or motivational factors associated with CSA practices including improved crop yields (OR=10.356; p < 0. 001) and reduced farm input costs (OR=4.855; p < 0.05) were significantly more associated with the adoption of CSA in the study context. In model 3, after controlling for support factors for CSA, age, educational attainment, marital status, and benefits or motivational factors associated with CSA practices such as improved crop yields remained significant (see Table 4). In addition to that, farmers who received adequate support from agricultural extension officers (OR=10.758; p < 0. 001) were significantly more likely to adopt CSA compared to those who did not receive any support from the extension officers. Furthermore, farmers who received training or education on CSA practices (OR=4.374; p < 0. 05) were more likely to adopt CSA practices on their farms than those who received no training. Interestingly, however, those who received adequate financial support (OR=0.205; p < 0.1) significantly reported lower odds of CSA adoption in the study context. Based on model fitness, model three is more robust with a R^2^ value of 0.5108, and the discussion was based on model three.

**Table 3:** Bivariate logistic regression results of the adoption of CSA practices.

Variables	OR(SE)	[95% CI]
**Variables**		
**Age (ref: 18–25)**		
26-35	1.164 (0.911)	0.251–5.400
36-45	0.606 (0.484)	0.126–2.903
46-59	1.778 (2.234)	0.151–20.863
60+	0.741 (0.948)	0.060–9.094
**Gender (ref: Male)**		
Female	1.164 (0.501)	0.501–2.707
**Education: (ref: No Formal Education)**		
Formal Education	9.86 (4.285) ***	4.207–23.111
**Marital Status (ref: Never Married)**		
Married	4.363 (1.867) ***	1.886–10.092
Widowed/Divorced	1.951 (2.181)	0.218–17.448
**My Practice of CSA has Improved my crop yields (ref: No)**		
Yes	29.210 (13.351) ***	11.925–71.545
**My practice of CSA has increased the resilience of my Farm (ref: No)**		
Yes	2.259 (0.885)	1.048–4.869
**My practice of CSA has reduced my input costs** **(e.g., fertilizer) (ref: No)**		
Yes	15.293 (6.489) ***	6.657–35.129
**I receive adequate support from agricultural extension officers (ref: No)**		
Yes	3.716 (1.658) **	1.549–8.911
**There are sufficient financial** **incentives (ref: No)**		
Yes	1.256 (0.522)	0.556–2.838
**I have received training or education on CSA practices. (ref: No)**		
Yes	10.184 (4.168) ***	4.567 (22.712)
**Government Policies (ref: No)**		
Yes	1.842 (0.789)	0.795–4.268
**I have the necessary tools and machinery for CSA practices (ref: No)**		
Yes	1.171(0.487)	0.518–2.645
**I have access to subsidies for the implementation of CSA (ref: No)**		
Yes	1.116 (0.464)	0.493–2.522

***p < 0.001, **p < 0.01, *p < 0.05, P < 0.1: OR: odds ratio, SE: standard error: CI: confidence interval.

**Table 4:** Multivariate results logistic regression results of the adoption of CSA practices.

Variables	Model 1		Model 2		Model 3	
**Age (ref: 18–25)**	**OR(SE)**	**[95% CI]**	**OR(SE)**	**[95% CI]**	**OR(SE)**	**[95% CI]**
26-35	0.305 (0.283)	0.049–1.881	0.102 (0.141) *	0.007–1.528	0.078 (0.126)	0.003–1.837
36-45	0.425 (0.422)	0.061–2.978	0.111 (0.156)	0.007–1.746	0.050 (0.083) *	0.002–1.298
46-59	1.246 (1.782)	0.076–20.527	0.267 (0.472)	0.008–8.531	0.124 (0.257)	0.002–7.249
60+	1.326 (1.894)	0.081–21.789	0.807 (1.502)	0.021–31.009	1.031 (2.331)	0.012 86.768
**Gender (ref: Male)**						
Female	1.578 (0.795)	0.588–4.236	1.176 (0.683)	0.376–3.672	1.449 (0.949)	0.401 5.237
**Education: (ref: No Formal Education)**						
Formal Education	15.785 (8.365) ***	5.587–44.597	6.814 (4.139) **	2.071–22.414	9.262 (6.562) ***	2.310 37.137
**Marital Status (ref: Never Married)**						
Married	6.508 (3.618) ***	2.188–19.348	4.476 (3.235) **	1.086–18.451	5.037 (4.315) *	0.939 27.004
Widowed/Divorced	3.727 (4.898)	0.284–48.99	7.376 (12.051)	0.300–181.343	11.742 (26.189)	0.148 929.531
**CSA Improve my crop yields (ref: No)**						
Yes			10.356 (6.559) ***	2.993–35.833	13.325 (10.298) ***	2.929 60.610
**CSA increase the resilience of my Farm (ref: No)**						
Yes			0.642 (0.362)	0.212–1.938	0.617 (0.407)	0.169–2.250
**CSA reduces input costs** **(e.g., fertilizer) (ref: No)**						
Yes			4.855 (2.689) **	1.639–14.381	4.862 (3.116)	1.385–17.076
**I receive adequate support from agricultural extension officers (ref: No)**						
Yes					10.758 (7.641) ***	2.674–43.279
**There are sufficient financial** **incentives (ref: No)**						
Yes					0.205 (0.169) *	0.041–1.027
**I have received training or education on CSA practices. (ref: No)**						
Yes					4.374 (2.824) **	1.234–15.504
**Government Policies (ref: No)**						
Yes					1.011 (0.829)	0.203–5.049
**I have access to subsidies for the implementation of CSA (ref: No)**						
Yes					0.339 (0.227)	0.092 (1.257)
**Goodness of fit measures**	R^2^ = 0.4052, Number of observations = 400		R^2^ = 0.4411, Number of observations = 400		R^2^ = 0.5108, Number of observations = 400	

***p < 0.001, **p < 0.01, *p < 0.05, OR: odds ratio, SE: standard error: CI: confidence interval.

## 5. Discussion

This study examines factors influencing CSA adoption in semi-arid Ghana using three nested regression models. The findings showed that CSA adoption is associated with age, with farmers aged 26–35 and 36–45 less likely to adopt CSA practices. This is probably because these age groups may be employed in the formal sector, or may be interested in seeking white-collar jobs rather than engaging in agriculture. This has negative implications for food production and security in general, as a very youthful and energetic age group, 26–35, is among the farmers, who are less likely to adopt CSA. Our finding is not peculiar to the UWR. [25] did not find an association between age and CSA adoption practices. However, our finding is contrary to those of Kalu and Mbanasor (2023) [26] and Gudina and Alemu (2024) [27], which showed that farmers’ age influences their decision to adopt CSA practices. The finding that education is a significant factor influencing CSA adoption by farmers suggests that those with some form of formal education were more likely to adopt CSA, due to their ability to understand and evaluate the relative importance of CSA. This can be associated with exposure to climate information and the ability to comprehend the benefits of adopting CSA practices and the risks of not adopting them [27]. This is consistent with the findings of Antwi and Antwi-Agyei [30], which show that education influences the adoption of various CSA technologies in the rice landscape in Mali.

Marital status is a strong predictor of CSA adoption, which could be explained by the fact that married farmers benefit from joint decision-making, in which couples pool ideas, resources, and labour to engage in CSA. This is consistent with Ajzen’s (1991) [12] conception of subjective norms, whereby married couples may encourage one another to adopt CSA. In the Upper East Region of Ghana, Antwi & Antwi-Agyei (2023) also found that married smallholder farmers employed CSA interventions on their farms. [29] and Wrigley-Asante et al. (2019) [31] attributed the association to household consumption sizes and the need to diversify crop production to increase food production and sustain large households, as well as to increase income from their farms. The association is further attributed to ease of access to land and the security of land tenure, particularly in areas where land is owned by families, as in the study area. Atta-Aidoo and Antwi-Agyei (2025) explained that marriage serves as a means of accessing land in sub-Saharan Africa, given that families hold the majority of land. Nonetheless, [32] working in southern Ethiopia, did not find any association between marital status and CSA adoption. This is likely due to gender dynamics in which women’s ability to make farming decisions may depend on cultural or societal norms, regardless of their marital status. The strong association and the explanations have positive implications for food security, livelihoods sustainability, and poverty reduction in the study area if an enabling environment is created and sustained for CSA adoption.

The relationship between motivational factors and farmers’ decisions to adopt CSA practices revealed that farmers who experienced improved crop yields through CSA practices were more likely to adopt CSA practices than those who did not. In the context of the Theory of Planned Behaviour, farmers may weigh the benefits of adopting CSA when deciding to adopt CSA. Farmers’ attitudes toward CSA adoption may be influenced by observed crop yields among other farmers who have adopted CSA strategies. For instance, [33] showed that farmers’ CSA adoption packages directly increase agricultural productivity, thereby enhancing crop yields and reducing vulnerability to climate change. CSA adoption facilitates greater plant uptake of essential nutrients, which is necessary for increased crop yields [34]. Similarly, a study by [35] shows that CSA practices significantly increased maize yield. Seeing these outcomes among farmers who practice CSA may encourage other farmers to adopt CSA practices.

The results of our study also show that respondents whose CSA practices reduced their farm input costs were more likely to adopt CSA than those with higher input costs. This is consistent with the theory of planned behaviour, which posits that a person is influenced by an action they believe to be delightful or unpleasant and may adopt it with an affective attitude. In the context of CSA adoption, farmers may weigh the benefits of reduced input costs against the costs of adopting CSA [15]. CSA practices help reduce farmers’ need to purchase chemical fertilizers by naturally enhancing soil fertility over time. CSA also improves water-use efficiency, reducing irrigation costs and the need for farmers to spend on irrigation technologies [30].

Our evaluation of the relative importance of the support farmers receive in their quest for CSA indicates that adequate support from agricultural extension officers was significantly associated with CSA adoption practices. This is consistent with the theory of planned behaviour, which defines subjective norm as the support provided by significant others for undertaking a particular activity. Subjective norms can be injunctive, where others encourage an individual to participate in an activity. In the context of CSA, farmers may consider seeking support from extension officers to adopt CSA. If extension officers highlight the significance of CSA, farmers are more likely to embrace information, training, and resources that support their adoption of these practices [18].

This is consistent with the findings of [36], who found that contact with extension agents has a statistically significant and positive effect on the level of CSA adoption practices in South Africa. In Ghana, the MoFA deploys extension officers to various communities to assist smallholder farmers in their farming practices. The government uses them as a conduit to disseminate information, render training on new technologies, and distribute farm inputs such as fertilizers and improved seeds. Farmers can ask them questions about their farming practices and how to increase crop yields. In the literature, agricultural extension services have been proven to build farmers’ agricultural knowledge and skills, disseminate new technologies, and change farmers’ attitudes toward evolving farming practices [37]. In CSA practice, studies have shown that farmers who interact frequently with agricultural extension officers have access to the agricultural information and modern farming technologies required for CSA adoption. This is consistent with findings by [25] and [38], who reported that frequent contact with agricultural officers may increase CSA adoption.

In addition, receiving sufficient financial relief was significantly associated with CSA adoption. Incentives such as subsidies and access to credit from microfinance institutions are important for CSA adoption. It allows farmers to allocate a budget for CSA adoption [39]. As explained by Ajzen (1991) [12], perceived behavioural control refers to people’s perceptions of how easy or difficult it is to perform the activity of interest. It also tells how capable and confident an individual is in performing a particular behaviour. Individuals who believe they lack the resources or opportunities to engage in a specific behaviour are unlikely to have strong intentions to do so (Kan & Fabrigar, 2017) [19]. In the context of CSA adoption, perceived behavioural control can significantly influence farmers’ intentions and actual adoption. If farmers perceive they have access to the necessary resources to implement CSA, their perceived behavioural control will be higher, increasing the likelihood of adoption. Conversely, if they perceive a lack of resources, their confidence may diminish, reducing the likelihood of adopting CSA. Many CSA practices, such as conservation tillage, improved irrigation systems, agroforestry, and the adoption of drought-resistant crop varieties, are needed in a semi-arid region like the Upper West Region and require sufficient financial incentives to cushion farmers. Farmers who lack access to credit and subsidies are less likely to adopt CSA practices [40]. Studies by Njogu et al. (2024) [35] and Adebisola & Ayodeji (2022) [40] show that access to credit significantly and positively affects smallholder farmers’ CSA adoption. As expected, farmers who received some training or education on CSA practices were significantly more likely to adopt CSA practices. As explained by the theory of planned behaviour, a support system, such as extension services and training programs, can enhance farmers’ perceived behavioural control. When farmers know they can seek help or guidance, they are more likely to feel capable of adopting CSA practices.

This is consistent with the findings of [41] and contrary to those of Chicas et al. (2023) [42], who found that farmers who received training were less likely to adopt CSA practices. Access to training, particularly CSA training, is one key determinant of CSA adoption practices among smallholder farmers. These trainings provide farmers with the knowledge and skills to implement CSA practices. According to [41], training plays a crucial role in exposing farmers to the supportive institutions, input sources, and credit facilities necessary for implementing CSA. Studies by other researchers also emphasize the importance of relevant policies that support CSA adoption and facilitate market access, enabling smallholder farmers to fully profit from their produce [11,43]. Despite its important findings, this study is not without limitations. First, the study tested only associations, not causal relationships. The selection of only communities that have been exposed to CSA practices is acknowledged as a limitation, although not all farmers in these communities were exposed to CSA interventions. This limitation could introduce bias into the sample and inflate the CSA adoption rate. Thus, the study’s findings should not be generalized to include communities that have not been exposed to CSA interventions.

## 6. Conclusion and policy recommendations

This study has highlighted the significant role of institutions in influencing CSA adoption through agricultural education and training programs using demonstration farms and farmer field schools. As a result, the study concluded that to achieve effective CSA adoption among smallholder farmers, existing institutions must be strengthened to implement relevant policies that support its adoption. It is crucial to foster synergies among the government, the private sector, and non-governmental organizations (NGOs) to establish an effective institutional framework that supports and promotes CSA in the agricultural sector. Similarly, supportive programmes and policy interventions should emphasize CSA adoption. To boost domestic productivity, policymakers should encourage and provide technical assistance, such as simple machinery, to smallholder farmers who adopt CSA practices that promote soil fertility and productivity. However, given that this research was time-limited and budget-constrained, it should be replicated across multiple locations to provide policymakers and practitioners with a clearer understanding of the factors influencing CSA adoption. Based on the findings, policymakers and non-governmental organizations (NGOs) that promote CSA practices should strengthen financial support systems and extension services for farmers, and provide more training on CSA. Relevant stakeholders should also consider the socio-demographic characteristics of smallholder farmers when promoting CSA.

In response to the challenges posed by global climate change, farming operations are now more successful due to CSA. A few studies have examined the factors affecting the level of CSA adoption among smallholder farmers in Semi-Arid Ghana. This study closes that gap by examining the factors influencing the extent to which smallholder farmers in the UWR of Ghana adopt climate-smart agricultural practices. Farmers in the study area generally practice crop diversification, water conservation, the use of improved seeds, and soil fertility management (crop residues, animal manure, and compost). The study found that education, age, marital status, improvements in crop yields, reductions in input costs, support from extension officers, financial support, and education/training were factors associated with CSA adoption among farmers in the Upper West Region (UWR).

## Supporting information

S1 File Inclusivity in global research. (DOCX)
