# Supplementary material for: Challenges to the adoption of climate-smart agricultural practices among smallholder farmers in semi-arid Ghana
Source: PLoS One. 2026 Aug 13;21(8):e0355679. doi: 10.1371/journal.pone.0355679 (PMC13472385; doi:10.1371/journal.pone.0355679)
Supplement: Supplementary file 1 [file pone.0355679.s003.pdf]

PLOS ONE

Adoption of Climate-Smart Agricultural Practices Among Smallholder Farmers in Semi-Arid Ghana.

--Manuscript Draft--

**Manuscript Number:**	PONE-D-25-35144
**Article Type:**	Research Article
**Full Title:**	Adoption of Climate-Smart Agricultural Practices Among Smallholder Farmers in Semi-Arid Ghana.
**Short Title:**	Climate-Smart Agricultural Practices Among Smallholder Farmers in Ghana.
**Corresponding Author:**	Mildred Molle, MA<br>University of Western Ontario: Western University<br>London, Ontario CANADA
**Keywords:**	Climate-smart agriculture; CSA practices; CSA adoption; smallholder farmers
**Abstract:**	With the increasing recognition of climate change threats to agricultural productivity and food security, it is crucial to explore farming practices that increase productivity, adapt to the effects of global climate change while lowering greenhouse gas emissions. This study investigates the factors that influence the adoption of climate-smart agricultural (CSA) practices among smallholder farmers in semi-arid Ghana specifically, the Upper West Region (UWR) of Ghana.<br>The study surveyed 400 smallholder farmers from eleven (11) Communities and employed stratified random sampling, and logistic regression model for data analysis. The results revealed that knowledge of CSA benefits or motivation factors such as improved crop yields (OR=10.356; p<0. 001), reduced input cost (OR=4.855; p<0.05), influenced the decision of smallholder farmers to adopt CSA practices. Also, availability of support systems such as access to agricultural extension services (OR=10.358; p<0. 001) and CSA training (OR=4.374; p<0. 05) were significantly associated with the adoption of CSA practices. Moreover, the study indicated a significant relationship between demographic factors such as age, education, and marital status of farmers and their decision to adopt CSA practices. Based on the findings, improved crop yields, resilience, reduced input, support from AEAs, financial support, training, and government policies are factors that influence CSA adoption among smallholder farmers in the study area.
**Order of Authors:**	Juliana Toboyee
	Mildred Naamwintome Molle
	Kenneth Peprah
	Raymond Aabeyir
	Sulemana Ansumah Saaka
	Isaac Luginaah
**Additional Information:**	
**Question**	**Response**
**Financial Disclosure**<br><br>Enter a financial disclosure statement that describes the sources of funding for the work included in this submission. Review the submission guidelines for detailed requirements. View published research articles from *PLOS ONE* for specific examples.	The author(s) received no specific funding for this work.

Powered by Editorial Manager® and ProduXion Manager® from Aries Systems Corporation

This statement is required for submission and **will appear in the published article** if the submission is accepted. Please make sure it is accurate.<br><br>**Funded studies**<br>Enter a statement with the following details:<br>• Initials of the authors who received each award<br>• Grant numbers awarded to each author<br>• The full name of each funder<br>• URL of each funder website<br>• Did the sponsors or funders play any role in the study design, data collection and analysis, decision to publish, or preparation of the manuscript?<br><br>Did you receive funding for this work?	
**Competing Interests**<br><br>Use the instructions below to enter a competing interest statement for this submission. On behalf of all authors, disclose any competing interests that could be perceived to bias this work—acknowledging all financial support and any other relevant financial or non-financial competing interests.<br><br>This statement is required for submission and **will appear in the published article** if the submission is accepted. Please make sure it is accurate and that any funding sources listed in your Funding Information later in the submission form are also declared in your Financial Disclosure statement.<br><br>View published research articles from *PLOS ONE* for specific examples.	The authors have declared that no competing interests exists

**NO authors have competing interests**<br><br>Enter: *The authors have declared that no competing interests exist.*<br><br>**Authors with competing interests**<br><br>Enter competing interest details beginning with this statement:<br><br>*I have read the journal's policy and the authors of this manuscript have the following competing interests: [insert competing interests here]*<br><br>* typeset	
**Ethics Statement**<br><br>Enter an ethics statement for this submission. This statement is required if the study involved:<br><br>• Human participants<br>• Human specimens or tissue<br>• Vertebrate animals or cephalopods<br>• Vertebrate embryos or tissues<br>• Field research<br><br>Write "N/A" if the submission does not require an ethics statement.<br><br>General guidance is provided below. Consult the submission guidelines for detailed instructions. **Make sure that all information entered here is included in the Methods section of the manuscript.**	The Simon Diedong Dombo University of Business and Integrated Development Studies (SDD UBIDS) Research Ethics Review Board granted ethical approval for the study (UBIDS/RERB/002/2025/02/04). The study obtained written consent from the participants

Powered by Editorial Manager® and ProduXion Manager® from Aries Systems Corporation

**Format for specific study types**<br><br>**Human Subject Research (involving human participants and/or tissue)**<br>• Give the name of the institutional review board or ethics committee that approved the study<br>• Include the approval number and/or a statement indicating approval of this research<br>• Indicate the form of consent obtained (written/oral) or the reason that consent was not obtained (e.g. the data were analyzed anonymously)<br><br>**Animal Research (involving vertebrate animals, embryos or tissues)**<br>• Provide the name of the Institutional Animal Care and Use Committee (IACUC) or other relevant ethics board that reviewed the study protocol, and indicate whether they approved this research or granted a formal waiver of ethical approval<br>• Include an approval number if one was obtained<br>• If the study involved *non-human primates*, add *additional details* about animal welfare and steps taken to ameliorate suffering<br>• If anesthesia, euthanasia, or any kind of animal sacrifice is part of the study, include briefly which substances and/or methods were applied<br><br>**Field Research**<br><br>Include the following details if this study involves the collection of plant, animal, or other materials from a natural setting:<br>• Field permit number<br>• Name of the institution or relevant body that granted permission	
**Data Availability**<br><br>Authors are required to make all data underlying the findings described fully available, without restriction, and from the time of publication. PLOS allows rare exceptions to address legal and ethical concerns. See the PLOS Data Policy and FAQ for detailed information.	Yes - all data are fully available without restriction

A Data Availability Statement describing where the data can be found is required at submission. Your answers to this question constitute the Data Availability Statement and **will be published in the article**, if accepted.<br><br>**Important:** Stating 'data available on request from the author' is not sufficient. If your data are only available upon request, select 'No' for the first question and explain your exceptional situation in the text box.<br><br>Do the authors confirm that all data underlying the findings described in their manuscript are fully available without restriction?	
**Describe where the data may be found in full sentences. If you are copying our sample text, replace any instances of XXX with the appropriate details.**<br><br>• If the data are **held or will be held in a public repository**, include URLs, accession numbers or DOIs. If this information will only be available after acceptance, indicate this by ticking the box below. For example: *All XXX files are available from the XXX database (accession number(s) XXX, XXX.).*<br>• If the data are all contained **within the manuscript and/or Supporting Information files**, enter the following: *All relevant data are within the manuscript and its Supporting Information files.*<br>• If neither of these applies but you are able to provide **details of access elsewhere**, with or without limitations, please do so. For example:<br><br>*Data cannot be shared publicly because of [XXX]. Data are available from the XXX Institutional Data Access / Ethics Committee (contact via XXX) for researchers who meet the criteria for access to confidential data.*<br><br>*The data underlying the results presented in the study are available from (include the name of the third party*	All the data are contained within the manauscript

and contact information or URL).<br>• This text is appropriate if the data are owned by a third party and authors do not have permission to share the data.<br><br>* typeset	
Additional data availability information:	Tick here if the URLs/accession numbers/DOIs will be available only after acceptance of the manuscript for publication so that we can ensure their inclusion before publication.

Powered by Editorial Manager® and ProduXion Manager® from Aries Systems Corporation

Manuscript Click here to access/download;Manuscript;Manuscript.docx

Adoption of Climate-Smart Agricultural Practices Among Smallholder Farmers in Semi-Arid Ghana.

Juliana Toboyee [1 2], Mildred Naamwintome Molle[2*], Kenneth Peprah[1], Raymond Aabeyir[1], Suleman Ansumah Saaka[2], Isaac Luginaah [2]

[1] SD Dombo University of Business and Integrated Development Studies Wa, Upper West Region, Ghana

[2]Department of Geography and Environment, Western University, London, Ontario, Canada

*Corresponding author:mmolle@uwo.ca

All the authors contributed equally to this work.

Abstract

With the increasing recognition of climate change threats to agricultural productivity and food security, it is crucial to explore farming practices that increase productivity, adapt to the effects of global climate change while lowering greenhouse gas emissions. This study investigates the factors that influence the adoption of climate-smart agricultural (CSA) practices among smallholder farmers in semi-arid Ghana specifically, the Upper West Region (UWR) of Ghana.

The study surveyed 400 smallholder farmers from eleven (11) Communities and employed stratified random sampling, and logistic regression model for data analysis. The results revealed that knowledge of CSA benefits or motivation factors such as improved crop yields (OR=10.356; $p<0.001$), reduced input cost (OR=4.855; $p<0.05$), influenced the decision of smallholder farmers to adopt CSA practices. Also, availability of support systems such as access to agricultural extension services (OR=10.358; $p<0.001$) and CSA training (OR=4.374; $p<0.05$) were significantly associated with the adoption of CSA practices. Moreover, the study indicated a significant relationship between demographic factors such as age, education, and marital status of farmers and their decision to adopt CSA practices. Based on the findings, improved crop yields, resilience, reduced input, support from AEAs, financial support, training, and government policies are factors that influence CSA adoption among smallholder farmers in the study area.

Keywords: Climate-smart agriculture, CSA practices, CSA adoption, smallholder farmers

1.0 Introduction

Climate change is widely recognized as a significant global threat with far-reaching implications for development. For instance, according to Dokos & Afifi, [1]. Similarly, the Lancet report emphasized that climate change is currently the biggest threat to global health. [2] reiterated how climate change could impede development initiatives and worsen inequality. It is acknowledged that alterations in temperature, rainfall patterns, and other climatic parameters are occurring at an increasing rate [3]. Research indicates that the impacts of climate change, weather variations, and extreme environmental events vary across countries, with a consensus that poorer nations bear a disproportionately severe burden compared to wealthier ones [4,5]. For example, the average surface temperature of the Earth has increased by 0.11°F (0.06°C) every decade since 1850, for a total increase of approximately 2°F (1.1°C). But since 1982, the rate of warming has noticeably increased, rising by 0.36°F (0.20°C) faster each decade. The warmest year on record was 2023, with temperatures exceeding both the pre-industrial average (1850–1900) and the 20th century average by 2.43°F (1.35°C). Incredibly, the ten warmest years in recorded history occurred during the last ten years (2014-2023). This warming trend has been greatly influenced by human activity, with global temperatures rising by about 1.1°C (1.9°F) since the pre-industrial era. Temperatures rose at a rate of about 0.15 to 0.20°C per decade after 1975, when the majority of this increase took place [6] thus, resulting in several climatic effects on nations and livelihoods.

The IPCC predicts that climate change increases rural communities' exposure and vulnerability to a variety of pressures, including poverty, food insecurity, water scarcity, and land degradation [7]. These stressors have an impact on the livelihood assets, activities, and outcomes of various groups of people, particularly women and marginalized groups who frequently lack access to resources,

information, and decision-making power [8,9]. Climate change also affects the biophysical and socioeconomic elements that influence agricultural yields, including temperature, precipitation, CO_2 concentration, pests and diseases, and market prices [10]. Climate change has both positive and negative effects on crop yields depending on crop type, location, and level of adaptation, but the general trend is expected to be unfavorable, especially in low-latitude regions [11]. For instance, global agricultural production is significantly impacted by climate change, which is reflected in lower yields of important crops like sorghum, maize, and rice. These yield losses are caused by various factors, including changing precipitation patterns, extreme weather events, and rising temperatures [10,12].

Uncertainty in agricultural production is made worse by the trend of an increase in extreme weather events, which especially affects staple crops like maize, rice, and soybeans. Global yields could drop by 12–20% by the end of the century if adaptation measures are not taken [10]. Rising temperatures also cause thermal stress in livestock, which lowers their productivity due to things like decreased feed intake, slower growth rates, and poorer reproductive outcomes. Furthermore, livestock death rates rise in response to climate change-related extreme heat events and disease outbreaks [12]. Moreover, water availability for drinking and fodder is impacted by climate change, which influences livestock productivity and health. Due to erratic weather patterns, crop failures, and declining incomes, these climate-related risks present serious obstacles to rural livelihoods [12]. In the end, decreasing crop yields pose a threat to the world's food security, highlighting the pressing need for adaptation strategies to guarantee a steady supply of food [10]. Reduced agricultural productivity has a particularly negative economic impact on developing nations where agriculture is a major source of income.

In Ghana, agriculture is a critical sector of the economy, employing 45.38% of the total workforce and contributing to food security, poverty alleviation. However, it is one of the sectors adversely affected by climate change, as the majority of farmers depend on rain-fed agriculture [13]. According to [14], climate change has altered rainfall patterns and farming seasons in Ghana, making it difficult for farmers to predict and organize their agricultural activities. This has led to poor agricultural yields, more pest and disease outbreaks, and worse income and food insecurity for farmers [15]. Furthermore, climate change has had an impact on farmers' local culture and indigenous knowledge, requiring them to adapt to new techniques and technologies that may conflict with their traditional values and beliefs [14].

In an attempt to address notable challenges in the agricultural sector with regards to climate change, the introduction of CSA methods in Ghana like minimum tillage, intercropping, soil nutrient amendment, and drought-tolerant crops have demonstrated to boost soil carbon stocks, minimize water and fertilizer consumption, and improve crop productivity. As a result, CSA is a viable solution for addressing the multifaceted issues of climate change and food security in Ghana, Africa, and beyond.

The detrimental impacts of climate change on agriculture have prompted the emergence of programmes that support sustainable practices and one such practice is CSA [16]. These programmes highlight the significance of farmers implementing strategies that address climate change and guarantee food security. CSA is a strategy for increasing the resilience and productivity of agricultural systems while lowering greenhouse gas emissions and achieving socioeconomic benefits [7]. CSA supports smallholder farmers to enhance their income, food security, and market access.

[17] investigated the effects of CSA practices on smallholder farmers in southern Ethiopia, an area susceptible to climate change. According to the research, smallholder farmers who implemented CSA practices such as crop management, water conservation, and agroforestry realized an increase in average annual farm income per hectare of 20.30% when compared to those who did not. Furthermore, by increasing crop yields and diversifying production, CSA methods have a direct impact on food security. Belay et al. [17] also reported that those who adopted the CSA practices had better access to nutrient-dense food, as evidenced by significantly higher food consumption scores (ranging from 6.27 to 8.15) between adopters and non-adopters. Also, a crucial element that is impacted by the adoption of CSA practices is market access. Through CSA, smallholder farmers can participate in value chains, reach markets, and sell their produce, all of which support market-oriented agriculture. Transferring CSA knowledge, connecting farmers with markets, and offering technical assistance depend on efficient extension services. Also, CSA enables women and other marginalized groups to participate in decision-making, capacity building, and resource allocation. The benefits of CSA are well articulated in literature, but these benefits can only be realized if farmers adopt CSA practices. To support policymakers and farmers in decision making on adopting CSA, it is imperative to establish the factors that influence the adoption of CSA practices. The study therefore seeks to examine the factors that influence the adoption of CSA practices among smallholder farmers in semi-arid Ghana specifically, the Upper West Region of Ghana.

2.0 Theoretical context

This study draws theoretical insights from the theory of planned behavior [18]. The theory of planned behavior is commonly used in health studies to understand factors that influence individuals' decisions to undertake a particular activity such as self-breast examination and

tobacco/cigarette intake [19,20]. The useful application of this theory within the context of climate-smart agriculture will facilitate the understanding of factors influencing farmers' decision to adopt CSA practices on their farms. Our study drew on three constructs of the theory- attitudes, subjective norms, and perceived behavioral control [18].

Attitude is the degree to which a person views a certain behavior favorably or negatively. It is an individual's overall evaluation or thoughts about doing something. An attitude towards positive behavior increases the possibility of intention to perform it. Behavioral beliefs and outcome judgments make up attitude. It can also be divided into affective and instrumental attitudes. The person believes the action is delightful or unpleasant with an affective attitude, and the behavior is beneficial or detrimental with an instrumental attitude. In the context of CSA adoption, farmers may weigh the benefits which include enhancement of agricultural productivity, improved food security, increased farm resilience against climate change, and reduced input cost [21]. Subjective norm**,** the second construct is a social pressure to perform or not to perform a given behaviour [18,22]. The subjective norm is the support given by significant others to undertake a particular activity. Subjective norms can be injunctive (whether others encourage you to do the behaviour.) or descriptive (whether others in a person's social group engage in the behaviour) [23]. In the context of CSA, farmers may consider whether other farmers, family members or extension officers support or discourage the adoption of CSA practices. Farmers often look to their peers for guidance and validation. If they observe that other farmers in their community are successfully adopting climate-smart practices and receiving positive feedback or benefits (such as increased yields or improved soil health), they may be more inclined to follow suit. This peer influence can create an effect, where the adoption of CSA becomes a common practice within the community.

Additionally, subjective norms play a crucial role in the dissemination of information about CSA. If extension officers highlight the significance of CSA, farmers are more likely to embrace information, training, and resources that support their adoption of these practices [24].

The third construct perceived behavioral control [18], is important in the theory since it corresponds to people's perceptions of the ease or difficulty in conducting the activity of interest. Perceived behavioral control explains the capability and confidence an individual has in performing a particular behaviour [18]. Individuals who believe they have neither the resources nor opportunities to perform a certain behavior are unlikely to have high intentions to perform the behavior [25]. In the context of adopting CSA, perceived behaviour control can significantly influence farmers' intentions and actual adoption in several ways such as access to resources. If farmers perceive that they have access to the necessary resources to implement CSA, their perceived behaviour control will be higher, leading to a greater likelihood of adoption. Conversely, if they perceive a lack of resources, their confidence may diminish, reducing the likelihood of adopting CSA. If farmers also have a support system such as extension services, and training programs, this can enhance farmers' perceived behavioral control**.** When farmers know they can seek help or guidance, they are more likely to feel capable of adopting CSA practices. The theory of planned behavior offers a structured framework for comprehending the adoption of CSA.
It can be used to investigate the factors that influence the adoption of CSA practices among small-holder farmers in semi-arid Ghana.

3.1 Study context

The research was conducted in the UWR of Ghana. The UWR is a vast semi-arid area of 18,476 square kilometers located between longitudes 1° 36′ to 3° West and latitudes 9° 48′ to 11° North. *Fig. 1.* shows a map of the UWR. The UWR is marked by perineal bushfire, partly due to the culturally embedded practice of using fire for various overlapping livelihoods within the same area. This is key to the annual occurrence of wildfires and the associated damage to crops, which create both direct and indirect links to food insecurity for smallholder families [26]. The region features drought-resistant scattered trees, including *Eucalyptus sp.* (eucalyptus), *Faidherbia albida* (Acacia albida), *Mangifera indica* (mango), *Adansonia digitata* (baobab), *Blighia sapida* (ackee), *Parkia biglobosa* (dawadawa), *Anacardium occidentale* (cashew), and *Vitellaria paradoxa* (shea).

The UWR is part of Ghana's northern savannah ecological zone, where rainfed agriculture serves as the main source of income and livelihood [27]. This region is home to 901,502 residents, and its savannah landscape experiences a single rainy season along with a lengthy dry season known as harmattan, typical of West Africa. Daily temperatures in the area fluctuate between 21 °C and 40 °C, while annual rainfall ranges from 840 mm to 1,400 mm [27]. These climatic factors result in alterations to the cropping calendar for smallholder farmers, ultimately affecting agricultural productivity since most farming activities depend on rainfall.

Agriculture in the UWR is mostly subsistence-based, with smallholder farmers engaging in crop production and livestock rearing. However, the dependence on traditional farming methods and limited access to modern agricultural technology and irrigation facilities increase the vulnerability of farming communities [27]. The region is susceptible to extreme climate stressors that significantly impact agricultural productivity, poverty, and food insecurity, water insecurity, out-migration [28],

and environmental pollution [29]. The socioeconomic difficulties and limited access to credit and agricultural inputs further reduces the farmers' ability to adjust to changing climatic conditions (30). The farmer-to-agricultural extension agent (AEA) ratio is relatively low, with one AEA to more than 5000 farmers in the UWR [27]. Establishing direct one-on-one contact between AEA and farmers is quite difficult because of limited resources. These and many more render the UWR particularly vulnerable to climate change impacts. In this context, embracing CSA practices is essential for creating a supportive environment that promotes sustainable agriculture and helps endure climatic disturbances.

The UWR's culture is diverse, with various ethnic groups such as the Dagaaba, Waala, Sissala, and Brifo shaping the sociocultural dynamics in adopting new technologies, innovations, and agricultural practices. The study's significance lies in the UWR's reliance on agriculture and vulnerability to climate change. Also, this paper aligns with Inclusive Growth and Sustainable Development which is a key goal of agenda 2063 which aims to Eradicate poverty, achieve economic diversification, and ensure environmental sustainability.

It also aligns with the Sustainable Development Goals 2 which aims to end hunger, improve nutrition, and promote sustainable agriculture and goal 13 which seeks to take urgent action to combat climate change and its impacts.

Figure 1. *Map of the study area, Upper West*

4.0 Method

4.1 Data collection and sample

A cross-sectional design was utilized for the study. Stratified random sampling was used to select 400 smallholder farmers across the region from selected communities in the UWR of Ghana, where CSA is promoted and practiced. All the study communities (Busa, Buka, Bugbelle, Loggu, Tuori, Chaangu, Chapuri, Tolibr, Sentu, Gozir, and Silbelle) had received some kind of intervention from organizations that encourage the practice of CSA, and this was one of the criteria for selecting those communities. The survey instrument was designed in the Kobo Collect Application and was pre-tested for validity and reliability before administration. Additionally, only respondents aged 18 years or older were included in the study. The survey collected information on demographic, ecological, environmental, and institutional factors relevant to CSA adoption. A letter of consent was read out to respondents for them to either thumbprint or sign to provide consent to commence the study. This study complied with the Declaration of Helsinki, ensuring that we sought informed consent and that participants were aware of the benefits and risks of the study. Participants' recruitment started from 7th February 2025 to 19th February 2025, and data collection was done between February 24th to April 10th across all the communities selected for the study. The Simon Diedong Dombo University of Business and Integrated Development Studies (SDD UBIDS) Research Ethics Review Board granted ethical approval for the study (UBIDS/RERB/002/2025/02/04).

4.2 Measures

The dependent variable for this study is the adoption of CSA. In the survey, respondents were asked if they practice CSA. Responses to this question were coded as 1=yes and 2=no. Guided by both the theoretical framework of the study and existing literature; contextually important covariates were included in the analyses of the factors influencing adoption of CSA practices among smallholder farmers in UWR of Ghana. These variables included *demographics* such as

age, gender marital status, and educational attainment; *CSA benefits or motivational factors* such as improvement of crop yields, farm resilience, and reduction of input cost; as well as *support systems* such as adequate support from extension officers, sufficient financial initiatives, education or training on CSA practices, government policies, and access to subsidies for the implementation of CSA. These variables were coded as follows: Age (18-25, 26-35, 36-45, 46-59 and 60+), marital status (0=Never Married, 1=Married, 2= Widowed/Divorced), education (0=No formal education, 1= Formal Education), Improvement of Crop yields (1=Yes, 0=No), Farm resilience (1=Yes, 0=No), and reduction of input cost (1=Yes, 0=No), adequate support from extension officers (1=Yes, 0=No), sufficient financial initiatives (1=Yes, 0=No), education or training in CSA practices (1=Yes, 0=No), government policies (1=Yes, 0=No), and access to subsidies for the implementation of CSA (1=Yes, 0=No).

4.3 Analytical approach

Descriptive statistics, bivariate, and multivariable analyses were conducted in this study. A descriptive statistic is used to provide an overview of the study population. Also, bivariate analyses were conducted to understand the relationship between each predictor variable and the outcome variable CSA. Finally, multivariable logistic regression models were employed for multivariable analyses, to explore the collective relationship between the predictors and the outcome variables (i.e., CSA). Results from the regression models are reported in Odds Ratios (OR). Where (OR>1), farmers are more likely to adopt CSA practices, and with (OR<1, farmers are less likely to adopt CSA practices. (OR=1) indicates that CSA adoption is equally likely to occur in both situations. All statistical analyses were performed using STATA 18.0 software.

5.0 Results

5.1 Demographic characteristics of study participants

Table 1 presents the demographic characteristics and CSA practices of the respondents. About 92.75% of the respondents indicated to have adopted CSA practices on their farms. Out of the total respondents, 69.50% were males whiles 30.50% were females. In terms of education, 75.25% of the respondents had some formal education which could be basic education, secondary or tertiary education. Most of the respondents (85%) were married. In terms of the benefits of CSA, 92.25% of respondents indicated that the practice of CSA lead to improved crop yields and 60% indicated that CSA improves climate change resilience of their farm crops. Also, 85% of respondents indicated that CSA help in reducing associated input costs for their farms. In terms of support for CSA implementation, 52% of respondents received support from extension agents for CSA practices. Moreover, 86% of the respondents specified that they have received some form of training or education on CSA practices. Only 40.25% of respondents showed that there are government policies that encouraged the implementation CSA practices for their farms. Likewise, only 35.75% indicated that there is sufficient financial support for CSA implementation practices and only 33.25% have access to subsidies for the implementation of CSA.

Table 1. Descriptive Statistics of study participants

Variable	Frequency (%)
Adoption of CSA	
Agree	371(92.75)
Disagree	29 (7.25)
Age	
18-25	29 (7.25)
26-35	234 (58.50)
36-45	101 (25.25)
46-59	25 (6.25)
60+	11 (2.75)
Gender	
Male	278 (69.50)
Female	122 (30.50)
Education	
No Formal Education	99 (24.75)
Formal Education	301 (75.25)
Marital Status	
Never Married	51(12.75)
Married	340 (85.00)
Widowed/Divorced	9 (2.25)
My Practice of CSA has improved my crop yields	
No	31 (7.75)

Yes	369 (92.25)
My Practice of CSA has improved the resilience of my farm	
No	160 (40.00)
Yes	240 (60.00)
My Practice of CSA has reduced input cost	
No	60 (15.00)
Yes	340 (85.00)
I receive adequate support from extension officers	
No	192 (48.00)
Yes	208 (52.00)
There are sufficient financial initiatives	
No	257 (52.00)
Yes	143 (35.75)
I have received education or training in CSA practices	
No	56 (14.00)
Yes	344 (86.00)
Government Policies	
No	239 (59.75)
Yes	161 (40.25)
I have access to subsidies for the implementation of CSA	
No	267 (66.75)
Yes	133 (33.25)

5.2 Bivariate analyses of factors influencing the adoption of CSA

Table 2 presents the results for bivariate analyses. At the bivariate level, smallholder farmers with formal education (OR=9.86; p<0. 001) were significantly more likely to adopt CSA practices than those without formal educational attainment. Also, those who were married (OR=4.363; p<0. 001) were significantly more likely to adopt CSA. Furthermore, benefits of CSA or motivational factors such as improved crop yields (OR=29.210; p<0. 001) and reduced input costs (OR=15.293; p<0. 001) were significantly more associated with the adoption of CSA in the study context. Respondents who received adequate support from agricultural extension officers (OR=3.716; p<0. 05) were also more likely to adopt CSA practices than those who did not receive such support. Moreover, farmers who received training or education on CSA practices (OR=10.184; p<0. 001) were more likely to adopt CSA practices on their farms.

Table 1: Bivariate logistic regression results of the adoption of CSA practices

**Variables**	**OR(SE)**	**[95% CI]**
**Variables**		
Age (ref: 18-25)		
26-35	1.164(0.911)	0.251 5.400
36-45	0.606(0.484)	0.126 2.903
46-59	1.778(2.234)	0.151 20.863
60+	0.741(0 .948)	0.060 9.094
Gender (ref: Male)		
Female	1.164(0.501)	0.501 2.707
Education: (ref: No Formal Education)		

Formal Education	9.86(4.285) ***	4.207	23.111
Marital Status (ref: Never Married)			
Married	4.363(1.867) ***	1.886	10.092
Widowed/Divorced	1.951(2.181)	0.218	17.448
My Practice of CSA has Improved my crop yields (ref: No)			
Yes	29.210(13.351) ***	11.925	71.545
My practice of CSA has increased the resilience of my Farm (ref: No)			
Yes	2.259(0 .885)	1.048	4.869
My practice of CSA has reduced my input costs (e.g., fertilizer) (ref: No)			
Yes	15.293(6.489) ***	6.657	35.129
I receive adequate support from agricultural extension officers (ref: No)			
Yes	3.716(1.658) **	1.549	8.911
There are sufficient financial incentives (ref: No)			
Yes	1.256(0.522)	0.556	2.838
I have received training or education on CSA practices. (ref: No)			
Yes	10.184(4.168) ***	4.567(22.712)	

Government Policies (ref: No)		
Yes	1.842(0.789)	0.795 4.268
I have the necessary tools and machinery for CSA practices (ref: No)		
Yes	1.171(0.487)	0.518 2.645
I have access to subsidies for the implementation of CSA (ref: No)		
Yes	1.116(0 .464)	0.493 2.522
***p<0.001, **p<0.01, *p<0.05, P < 0.1: OR: odds ratio, SE: standard error: CI: confidence interval		

5.3 Multivariate analyses of factors influencing the adoption of CSA

The multivariate logistic regression results of the study are presented in a nested model format in Table 3. We controlled for demographic variables in Model 1, the benefits of CSA in Model 2, and the support available for the practices of CSA in Model 3. With the demographic covariates in Model 1, farmers with formal education (OR=15.785; p<0. 001) were more likely to adopt CSA practices on their farms compared to farmers with no formal education. Farmers who were married (OR=6.508; p<0. 001) were six times more likely to adopt CSA on their farms for greater farm output compared to those who are never married. Consistent with results in Model 1, in model 2, farmers with formal education (OR=6.814; p<0.05) as well as those who were married (OR=4.476; p<0.05) were six time and four times more likely to adopt CSA practices, respectively. However, while no significant association was found between age and CSA in Model 1, in Model 2, farmers

between the ages of 26-35 (OR=0.102; p<0.1) were more likely to adopt CSA practices. Also in Model 2, benefits or motivational factors associated with CSA practices including improved crop yields (OR=10.356; p<0. 001) and reduced farm input costs (OR=4.855; p<0.05) were significantly more associated with the adoption of CSA in the study context. In model 3, after controlling for support factors for CSA, age, educational attainment, marital status, and benefits or motivational factors associated with CSA practices such as improved crop yields remained significant (see Table 3). In addition to that, farmers who received adequate support from agricultural extension officers (OR=10.358; p<0. 001) were significantly more likely to adopt CSA compared to those who did not receive any support from the extension officers. Furthermore, farmers who received training or education on CSA practices (OR=4.374; p<0. 05) were more likely to adopt CSA practices on their farms than those who received no training. Interestingly, however, those who received adequate financial support (OR=0.205; p<0.1) significantly reported lower odds of CSA adoption in the study context. Based on model fitness, model three is more robust with a R^2 value of 0.5108, and the discussion was based on model three.

Table 2: Multivariate Results logistic regression results of the adoption of CSA practices

Variables	Model 1		Model 2		Model 3	
Age (ref: 18-25)	**OR(SE)**	**[95% CI]**	**OR(SE)**	**[95% CI]**	**OR(SE)**	**[95% CI]**
26-35	0.305(0.283)	0 .049 1.881	0.102(0.141) *	0.007 1.528	0.078(0.126)	0.003 1.837
36-45	0 .425(0.422)	0.061 2.978	0.111(0.156)	0.007 1.746	0.050(0.083) *	0.002 1.298
46-59	1.246(1.782)	0.076 20.527	0.267(0.472)	0.008 8.531	0.124(0.257)	0.002 7.249
60+	1.326(1.894)	0.081 21.789	0.807(1.502)	0.021 31.009	1.031(2.331)	0.012 86.768
Gender (ref: Male)						
Female	1.578(0 .795)	0.588 4.236	1.176(0.683)	0.376 3.672	1.449(0.949)	0.401 5.237
Education: (ref: No Formal Education)						

Formal Education	15.785(8.365) ***	5.587	44.597	6.814(4.139) **	2.071	22.414	9.262(6.562) ***	2.310 37.137
Marital Status (ref: Never Married)								
Married	6.508 (3.618) ***	2.188	19.348	4.476(3.235) **	1.086	18.451	5.037(4.315) *	0.939 27.004
Widowed/Divorced	3.727(4.898)	0.284	48.99	7.376(12.051)	0.300	181.343	11.742(26.189)	0.148 929.531
CSA Improve my crop yields (ref: No)								
Yes				10.356(6.559) ***	2.993	35.833	13.325(10.298) ***	2.929 60.610
CSA increase the resilience of my Farm (ref: No)								
Yes				0.642(0.362)	0.212	1.938	0.617(0.407)	0.169 2.250
CSA reduces input costs (e.g., fertilizer) (ref: No)								

Yes		4.855(2.689)** 1.639 14.381	4.862(3.116) 1.385 17.076
I receive adequate support from agricultural extension officers (ref: No)			
Yes			10.758(7.641)*** 2.674 43.279
There are sufficient financial incentives (ref: No)			
Yes			0.205(0.169)* 0.041 1.027
I have received training or education on CSA practices. (ref: No)			
Yes			4.374(2.824)** 1.234 15.504
Government Policies (ref: No)			

Yes			1.011(0.829) 0.203 5.049
I have access to subsidies for the implementation of CSA (ref: No)			
Yes			0.339(0.227) 0.092(1.257)
**Goodness of fit measures**	R^2=0.4052, Number of observations=400	R^2= 0.4411, Number of observations=400	R^2=0.5108, Number of observations=400
***p<0.001, **p<0.01, *p<0.05, OR: odds ratio, SE: standard error: CI: confidence interval			

6.0 Discussion

This study seeks to understand the factors that influence the adoption of CSA in semi-arid Ghana. The analysis was carried out in three-nested models where model one focused on socio-demographic factors and model two on both socio-demographic factors and benefits of CSA. The last model controlled for a combination of socio-demographic factors, the benefits of CSA, and the support systems available to support the adoption of CSA. With regards to demographic factors, the findings showed that the adoption of CSA is associated with age, whereby in models two and three, farmers between the ages of 26-35 and 36-45 were less likely to adopt CSA practices. This is probably due to the fact that these age groups are more formally educated and may be employed in white colour jobs or interested in seeking white color jobs rather than engaging in agriculture. This is contrary to the findings of Kalu & Mbanasor [31] and Gudina &Alemu [32] where the age of farmers influences their decision to adopt CSA practices. This study indicated that farmers within the young and Middle Ages were most industrious, energetic, and interested in new technologies, hence the decisions for CSA implementation. Nevertheless, contrary to the findings of this study, studies like [33] did not find an association between age and the adoption of CSA practices.

The emergence of education as a significant factor that influenced the adoption of CSA practices by farmers suggests that those with some form of formal education be it basic, secondary or tertiary were likely to adopt CSA due to their ability to understand and evaluate the relative importance of CSA with a positive attitude. This can be associated with exposure to climate information and their ability to comprehend information on the benefits and risks of not adopting CSA practices [32]. This is consistent with the findings of Sanogo et al. [34] where education influences the adoption

of various CSA technologies in the rice landscape in Mali. The emergence of marital status as a strong predictor of CSA adoption could be explained by the fact that a joint-decision making where married people pool ideas, resources, and labour to practice CSA. This is in line with [18] conception on subjective norms whereby married couples may encourage each other towards the adoption of CSA. Working in the Upper East Region of Ghana, Antwi & Antwi-Agyei [35] also found that both male and female married smallholder farmers employed CSA interventions on their farms. Antwi et al. [35] explained their findings as mostly due to the consumption sizes of their household and the need to diversify their crop production to include crop diversification, intercropping, and planting early maturing varieties of crops to help them increase food production to sustain their households and also increase income from their farms [35,36]. A similar positive association of marital status and adoption of CSA is reported by Adebisola & Ayodeji [37]. Nonetheless, Kassa et al. [38] working in southern Ethiopia did not find any association between marital status and CSA adoption. This is likely due to gender dynamics where women's ability to make farming decisions could depend on cultural or societal norms, regardless of their marital status.

The study further analyzed benefits and motivation factors and how they could influence farmers' decision to adopt CSA practices. Farmers who experienced improved crop yields through CSA strategies were more likely to adopt CSA practices compared to those who did not practice CSA. In the context of the theory of planned behaviour, farmers may weigh the benefits which include enhancement of agricultural productivity to adopt CSA. As stated in the theory, farmers attitude towards CSA adoption may be based on the improved crop yields of other farmers who have adopted the CSA strategies. For instance, Acevedo et al. [39] showed that farmers' adoption of

CSA packages directly increases agricultural productivity, thereby enhancing crop yields and reducing vulnerability to climate change. CSA adoption facilitates greater plant uptake of essential nutrients which is necessary for increased crop yields [40]. Similarly, a study by Akter et al. [40] shows that CSA practices significantly caused an increase in maize yield. Seeing these outcomes from farmers who practice CSA, other farmers may be encouraged to practice CSA strategies.

The results from our study also show that respondents whose CSA practices reduced their farm input costs were more likely to adopt CSA compared to those who still have greater input costs. This consistent with the theory of planned behaviour which projects that a person is influenced by an action which is believed to be delightful or unpleasant and may adopt with an affective attitude. In the context of CSA adoption, farmers may weigh the benefits of reduced input cost to adopt CSA [21]. CSA practices help reduce the need for farmers to purchase chemical fertilizers by enhancing soil fertility naturally as it is continually practiced. CSA also improves water-use efficiency, reducing irrigation costs and the need for farmers to spend on irrigation technologies [42].

Our evaluation of relative importance of the support farmers receive in their quest for CSA indicate that adequate support from agricultural extension officers was significantly associated with the adoption of CSA practices. This is consistent with the theory of planned behaviour that explains the subjective norm as the support given by significant others to undertake a particular activity. Subjective norms can be injunctive where others encourage an individual to participate in an activity. In the context of CSA, farmers may consider extension officers support for the adoption of CSA. If extension officers highlight the significance of CSA, farmers are more likely to embrace information, training, and resources that support their adoption of these practices [24].

This is consistent with the findings of Abegunde at al. [43] where contact with extension agents has a statistically significant and positive effect on the level of adoption of CSA practices in South Africa. In Ghana, the MoFA deploys extension officers to various communities to assist smallholder farmers in their farming practices. The government uses them as a conduit to disseminate information, render training on new technologies, and to distribute farm inputs such as fertilizers and improved seeds. Farmers get the chance to ask them questions about their farming practices and how to get increased crop yields. In the literature, agricultural extension services have been proven to build farmers' agricultural knowledge and skills, disseminate new technologies, and change farmers' attitudes toward evolving farming practices [44]. In the practice of CSA, studies have shown that farmers who have interactions and frequent contact with agricultural extension officers have access to agricultural information and modern farming technologies required for CSA technology adoption. This is in line with findings by Luu & Ndung'u et al. [33,45] who reported that frequent contact with agricultural officers has the potential to increase the adoption of CSA.

In addition, receiving sufficient financial relief was significantly associated with the adoption of CSA practices. Incentives such as subsidies, and access to credit from micro-finance institutions are important for the adoption of CSA. It allows farmers to have the budget for the adoption of CSA [46]. As explained by [18], perceived behavioral control informs people's perceptions of how easy or difficult it is to conduct the activity of interest. It also tells how capable and confident an individual is in performing a particular behaviour. Individuals who believe they have neither the resources nor opportunities to perform a certain behavior are unlikely to have high intentions to perform the behavior [25]. In the context of adopting CSA, perceived behaviour control can

significantly influence farmers' intentions and actual adoption. If farmers perceive that they have access to the necessary resources to implement CSA, their perceived behaviour control will be higher, leading to a greater likelihood of adoption. Conversely, if they perceive a lack of resources, their confidence may diminish, reducing the likelihood of adopting CSA. Many CSA practices such as conservation tillage, improved irrigation systems, agroforestry, or adopting drought-resistant crop varieties for a semi-arid location like the Upper West Region require sufficient financial incentives that farmers need to cushion them. Farmers who are unable to access credit and subsidies have lower chances of adopting CSA practices [37]. A study by Njogu et al. [47] and Adebisola et al. [37] shows that access to credit was significant and had a positive effect on smallholder farmers' adoption rates of CSA practices. As expected, farmers who received some training or education on CSA practices was significantly associated with the adoption of CSA practices. As explained by the theory of planned behaviour, if farmers have a support system such as extension services, and training programs, this can enhance farmers' perceived behavioral control**.** When farmers know they can seek help or guidance, they are more likely to feel capable of adopting CSA practices.

This is consistent with the findings of Nkuba et al. [48]. Contrary to the findings of this study, farmers who receive training in the study of Chicas et al. [49] were less likely to adopt CSA practices. Access to training, particularly CSA training, is one key determinant of the adoption of CSA practices among smallholder farmer. These trainings provide farmers with the knowledge and skills to implement CSA practices. According to Nkuba et al. [48], training plays a crucial role in exposing farmers to the supportive institutions, input sources, and credit facilities necessary for CSA implementation. Studies by other researchers also emphasize how crucial it is to have relevant

policies that support the adoption of CSAs and make connections with the market possible so that smallholder farmers can profit fully from their produce [17,50].

Despite its important findings, this study is not without limitations. First, the study only tested associations between the variables but not their causal relationships. Since the study adopted a cross-sectional design, causality cannot be deduced or established. Nevertheless, the findings have significant policy implications.

7.0 Conclusion and policy recommendations

This study has highlighted the significant role of institutions in influencing CSA adoption through agricultural education and training programs using demonstration farms and farmer field schools. As a result, the study concluded that to achieve any effective adoption of CSA among smallholder farmers, existing institutions must be strengthened to roll out relevant policies that support the adoption of CSA. It is crucial to foster synergies among the government, private sector, and non-governmental organizations (NGOs) in establishing an efficient institutional structure that supports and promotes CSA within the agricultural sector. Similarly, supportive programs, and policy intervention programs should emphasize the adoption of CSA practices. To boost domestic productivity, policymakers should encourage and provide technical assistance such as provision of simple machinery to smallholder farmers who adopt CSA practices that promote soil fertility and productivity. But for the fact that this research was time-limited and budget constrained, such research should be replicated at various locations to provide policymakers and practitioners a clearer comprehension of the factors influencing the adoption of CSA. Based on the findings, policymakers and non-governmental organizations (NGOs) that promote CSA practices should

enhance financial support systems and extension services to farmers, as well as provide more training on CSA. Relevant stakeholders should also take smallholder farmers' socio-demographic factors into account when promoting CSA.

In response to the challenges posed by global climate change, farming operations are now succeeding because of CSA. Few studies have been done to investigate the factors affecting the level of CSA adoption among smallholder farmers in Semi-Arid Ghana. This study closes that gap by examining the factors influencing the extent to which smallholder farmers in the UWR of Ghana, adopt climate-smart agricultural practices. Farmers in the study area generally embraced local CSA techniques like crop diversification, water conservation, use of improved seeds, and soil fertility management (crop residues, animal manure, and compost). The findings from the study revealed that education, age, marital status, improvement in crop yields, reduction in input cost, support from extension officers, financial support and education/training were factors associated with the adoption of CSA among farmers in the UWR.

Acknowledgement

We would like to express our sincere gratitude to all the participants for their time and contributions to the study. Their valuable insights have greatly shaped the outcome of this study and we are extremely grateful.

References

1. Dokos TP., Afifi Tamer. Climate change : addressing the impact on human security. Hellenic Foundation for European and Foreign Policy (ELIAMEP) : Hellenic Ministry of Foreign Affairs; 2008. 102 p.
2. Kotcher J, Maibach E, Miller J, Campbell E, Alqodmani L, Maiero M, et al. Views of health professionals on climate change and health: a multinational survey study. Vol. 5, The Lancet Planetary Health. Elsevier B.V.; 2021. p. e316–23.
3. Hoang HG, Tran DT. Vietnamese farmers' perception of climate change effects on water resources and determinants of climate change. Hydrological Sciences Journal. 2024;69(4):474–87.
4. Lipper L, Thornton P, Campbell BM, Baedeker T, Braimoh A, Bwalya M, et al. Climate-smart agriculture for food security. Vol. 4, Nature Climate Change. Nature Publishing Group; 2014. p. 1068–72.
5. Sadar A, Kiani AK, Kulsu Y, Bilgic A. Examining the Role of Livelihood Diversification as a Part of Climate-Smart Agriculture (CSA) Strategy. Atatürk Üniversitesi Ziraat Fakültesi Dergisi. 2020 Jan 25;79–87.
6. Lindsey RBY, Dahlman L. Vol. 18, https://www.energy.gov/. 2020. p. 2024 Climate Change: Global Temperature. Available from: http://www.climate.gov/media/15819
7. Birkmann J, Liwenga E, Pandey R. Poverty, Livelihoods and Sustainable Development. In: H.-O. Pörtner DC, Roberts M, Tignor ES, Poloczanska K, Mintenbeck A, Alegría M, et al., editors. Climate Change 2022 – Impacts, Adaptation and Vulnerability. Cambridge University Press; 2023. p. 1171–284.
8. Food and Agriculture Organization. Climate Change, Agriculture and Food Security [Internet]. 2013. Available from: www.fao.org/publications
9. Nelson S, Huyer S. Climate-smart agriculture A Gender-responsive Approach to Climate-Smart Agriculture Evidence and guidance for practitioners [Internet]. 2016 [cited 2025 Feb 1]. Available from: https://ccafs.cgiar.org/resources/tools/gender-responsive-approach-climate-smart-agriculture-evidence-and-guidance
10. Feng X, Tian H, Cong J, Zhao C. A method review of the climate change impact on crop yield. Vol. 6, Frontiers in Forests and Global Change. Frontiers Media SA; 2023.
11. Rezaei S, Buitink J, Hay FR. Contrasting seed moisture sorption behaviour between two species and the implication for seed longevity. Seed Sci Res. 2023 Jun 1;
12. Intergovernmental Panel on Climate Change. Chapter Climate Change 2014 Synthesis Report Summary for Policymakers Summary for Policymakers [Internet]. 2014 [cited 2025 Jan 29]. Available from: https://www.ipcc.ch/site/assets/uploads/2018/02/AR5_SYR_FINAL_SPM.pdf

13. Ministry of Food and Agriculture. Statistics Research, And Information Directorate of Ministry of Food And Agriculture [Internet]. 2022. Available from: http://www.srid.mofa.gov.gh

14. Boafo J, Yeboah T, Guodaar L, Stephanie Y, Nyantakyi-Frimpong H. Understanding non-economic loss and damage due to climate change in Ghana. Clim Dev. 2024;16(2):109–18.

15. Akudugu MA, Mahama ES, Akudugu MA, Dittoh S, Mahama ES. The Implications of Climate Change on Food Security and Rural Livelihoods: Experiences From Northern Ghana. 2012;2(3). Available from: www.iiste.org

16. Bebbington A. Capitals and Capabilities: A Framework for Analyzing Peasant Viability, Rural Livelihoods and Poverty. World Dev [Internet]. 1999; Available from: www.elsevier.com/locate/worlddev

17. Belay A, Mirzabaev A, Recha JW, Oludhe C, Osano PM, Berhane Z, et al. Does climate-smart agriculture improve household income and food security? Evidence from Southern Ethiopia. Environ Dev Sustain. 2024 Jul 1;26(7):16711–38.

18. Ajzen I. The Theory of Planned Behavior. Organ Behav Hum Decis Process [Internet]. 1991 [cited 2024 Dec 1]; Available from: https://doi.org/10.1016/0749-5978(91)90020-T

19. Norman P, Cooper Y. The theory of planned behaviour and breast self-examination: Assessing the impact of past behaviour, context stability and habit strength. Psychol Health. 2011 Sep;26(9):1156–72.

20. Topa G, Moriano JA. Theory of planned behavior and smoking: meta-analysis and SEM model. Subst Abuse Rehabil. 2010 Dec;23.

21. Sain G, Loboguerrero AM, Corner-Dolloff C, Lizarazo M, Nowak A, Martínez-Barón D, et al. Costs and benefits of climate-smart agriculture: The case of the Dry Corridor in Guatemala. Agric Syst. 2017 Feb 1;151:163–73.

22. Ham M, Jeger M, Ivković AF. The role of subjective norms in forming the intention to purchase green food. Economic Research-Ekonomska Istrazivanja . 2015;28(1):738–48.

23. Fishbein Martin, Ajzen Icek. Predicting and changing behavior : the reasoned action approach. Psychology Press, Taylor & Francis Group; 2010. 518 p.

24. Jha S, Singh S. Role of Agriculture Extension for Climate Smart Agriculture. In: Implications For Climate smart Agriculture [Internet]. Biotech Books; 2021. p. 199–208. Available from: https://www.researchgate.net/publication/353305266

25. Kan MPH, Fabrigar LR. Theory of Planned Behavior. In: Encyclopedia of Personality and Individual Differences. Springer International Publishing; 2017. p. 1–8.

26. Kpienbaareh D, Luginaah I. After the flames then what? exploring the linkages between wildfires and household food security in the northern Savannah of Ghana. International Journal of Sustainable Development and World Ecology. 2019;26(7):612–24.

27. Ministry of Food and Agriculture (MoFA). Agriculture In Ghana Facts and Figures (2018) [Internet]. 2019 [cited 2024 Feb 1]. Available from: https://mofa.gov.gh/site/publications/research-reports/376-agriculture-in-ghana-facts-figures-2018

28. Luginaah I, Weis T, Galaa S, Nkrumah MK, Benzer-Kerr R, Bagah D. Environment, Migration and Food Security in the Upper West Region of Ghana. In: Environment and Health in Sub-Saharan Africa: Managing an Emerging Crisis. Springer Netherlands; 2009. p. 25–38.

29. Antabe R, Atuoye KN, Kuuire VZ, Sano Y, Arku G, Luginaah I. To Move or not to Move: Community Members' Reaction to Surface Mining Activities in the Upper West Region of Ghana. Soc Nat Resour. 2020 Mar 3;33(3):368–85.

30. Batung ES, Mohammed K, Kansanga MM, Nyantakyi-Frimpong H, Luginaah I. Credit access and perceived climate change resilience of smallholder farmers in semi-arid northern Ghana. Environ Dev Sustain. 2023 Jan 1;25(1):321–50.

31. Kalu CA, Mbanasor JA. Factors Influencing the Adoption of Climate Smart Agricultural Technologies Among Root Crop Farming Households in Nigeria. FARA Research Report. 2023;7(57):744.

32. Gudina MH, Alemu EA. Factors influencing small holder farmers adoption of climate SMART agriculture practices in Welmera Woreda, Central Ethiopia. Frontiers in Climate. 2024;6.

33. Luu TD. Factors Influencing Farmers' Adoption of Climate-Smart Agriculture in Rice Production in Vietnam's Mekong Delta. Asian Journal of Agriculture and Development [Internet]. 2020 Jun 1;17(1):109–24. Available from: https://ajad.searca.org/read-articles/13-view-article?aid=1197

34. Sanogo K, Touré I, Arinloye DDAA, Dossou-Yovo ER, Bayala J. Factors affecting the adoption of climate-smart agriculture technologies in rice farming systems in Mali, West Africa. Smart Agricultural Technology. 2023 Oct 1;5.

35. Antwi K, Antwi-Agyei P. Intra-gendered perceptions and adoption of climate-smart agriculture: Evidence from smallholder farmers in the Upper East Region of Ghana. Environmental Challenges. 2023 Aug 1;12.

36. Wrigley-Asante C, Owusu K, Egyir IS, Owiyo TM. Gender dimensions of climate change adaptation practices: the experiences of smallholder crop farmers in the transition zone of Ghana. African Geographical Review. 2019 Apr 3;38(2):126–39.

37. Adebisola A, Ayodeji T. Factors Influencing Adoption of Climate Smart Agricultural Practices Among Maize Farmers In Ondo State, Nigeria. Journal of Economics and Allied Research. 2022;7(4).

38. Kassa BA, Abdi AT. Factors Influencing the Adoption of Climate-Smart Agricultural Practice by Small-Scale Farming Households in Wondo Genet, Southern Ethiopia. Sage Open. 2022 Jul 1;12(3).

39. Acevedo M, Pixley K, Zinyengere N, Meng S, Tufan H, Cichy K, et al. A scoping review of adoption of climate-resilient crops by small-scale producers in low- and middle-income countries. Nat Plants. 2020 Oct 1;6(10):1231–41.

40. Akter A, Geng X, Endelani Mwalupaso G, Lu H, Hoque F, Kiraru Ndungu M, et al. Income and yield effects of climate-smart agriculture (CSA) adoption in flood prone areas of Bangladesh: Farm level evidence. Clim Risk Manag. 2022 Jan 1;37.

41. Zizinga A, Mwanjalolo JGM, Tietjen B, Bedadi B, Gabiri G, Luswata KC. Impacts of Climate Smart Agriculture Practices on Soil Water Conservation and Maize Productivity in Rainfed Cropping Systems of Uganda. Front Sustain Food Syst. 2022 May 24;6.

42. Erekalo KT, Pedersen SM, Christensen T, Denver S, Gemtou M, Fountas S, et al. Review on the contribution of farming practices and technologies towards climate-smart agricultural outcomes in a European context. Smart Agricultural Technology. 2024 Mar 1;7.

43. Abegunde VO, Sibanda M, Obi A. Determinants of the adoption of climate-smart agricultural practices by small-scale farming households in King Cetshwayo district municipality, South Africa. Sustainability (Switzerland). 2020 Jan 1;12(1).

44. Zafarullah Khan M, Nawab K, Khatam A, Qasim M, Ayub G, Naima Nawaz and. Communication Gap and Training Needs Of Pakistan’s Agricultural Extension Agents In Horticulture. Vol. 28. 2012.

45. Ndung’u S, Ogema V, Thiga M, Wandahwa P. Factors Influencing the Adoption Of Climate Smart Agriculture Practices Among Smallholder Farmers In Kakamega. African Journal of Food, Agriculture, Nutrition and Development. 2023;23(10):24759–82.

46. Olutumise AI. Impact of credit on the climate adaptation utilization among food crop farmers in Southwest, Nigeria: application of endogenous treatment Poisson regression model. Agricultural and Food Economics. 2023 Dec 1;11(1).

47. Njogu JW, Karuku G, Busienei J, Gathiaka JK. Assessing determinants of scaling up pathways for adopted CSA Climate Smart Agricultural practices: Evidence from Climate Smart Villages in Nyando Basin, Kenya. Cogent Food Agric. 2024;10(1).

48. Nkuba MR, Chanda R, Mmopelwa G, Kato E, Mangheni MN, Lesolle D. Influence of Indigenous Knowledge and Scientific Climate Forecasts on Arable Farmers’ Climate

Adaptation Methods in the Rwenzori region, Western Uganda. Environ Manage. 2020 Apr 1;65(4):500–16.

49. Chicas SD, Nielsen JØ, Robinson GM, Mizoue N, Ota T. The adoption of climate-smart agriculture to address wildfires in the Maya Golden Landscape of Belize: Smallholder farmers' perceptions. J Environ Manage. 2023 Nov 1;345.

50. Abraham M, Pingali P. Transforming Smallholder Agriculture to Achieve the SDGs. In: The Role of Smallholder Farms in Food and Nutrition Security. Springer International Publishing; 2020. p. 173–209.

Figure

Click here to access/download;Figure;Figure_1- Map of study area.jpg

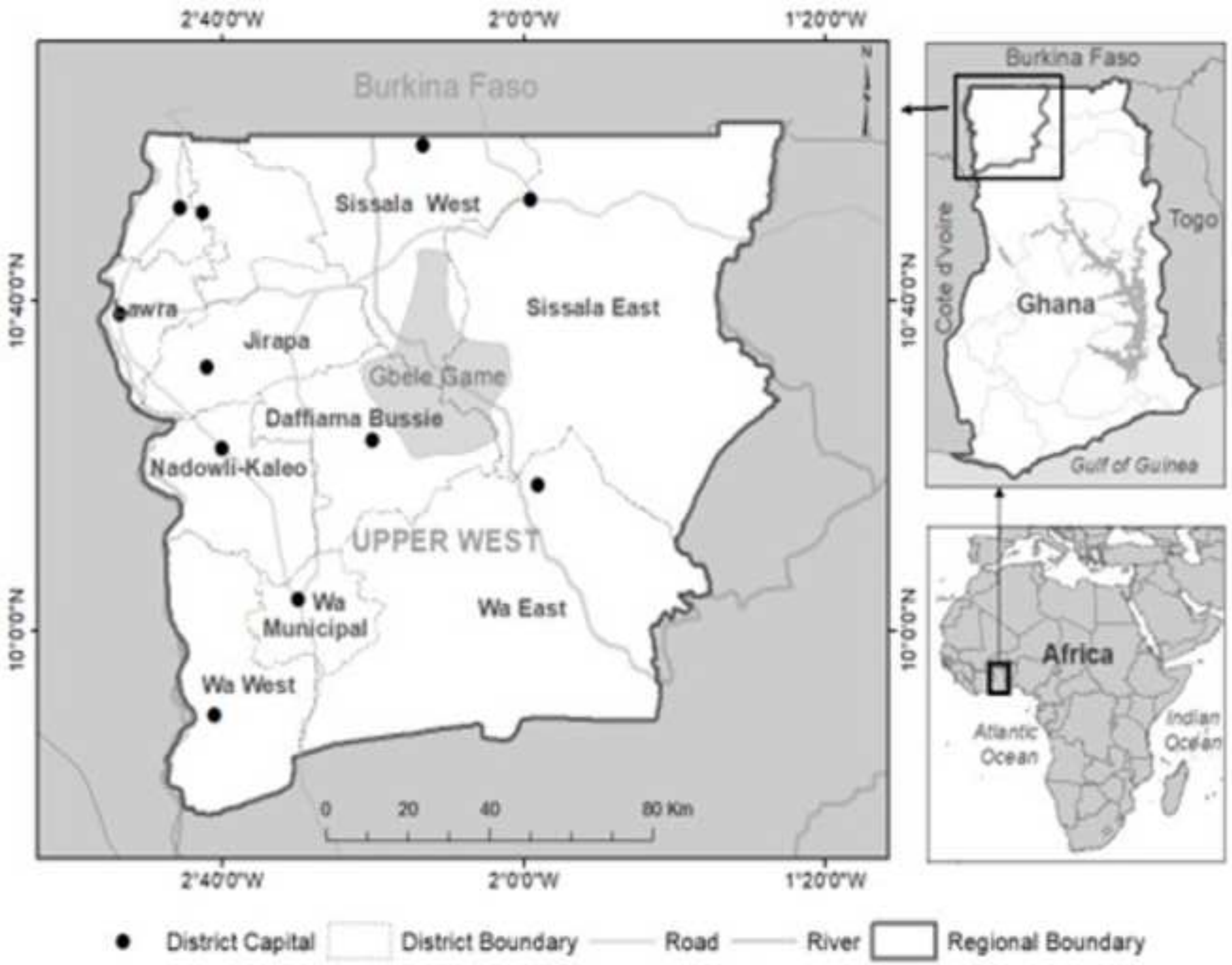

Click here to access/download
Supporting Information
Ethics.pdf
