# Supplementary material for: Challenges to the adoption of climate-smart agricultural practices among smallholder farmers in semi-arid Ghana
Source: PLoS One. 2026 Aug 13;21(8):e0355679. doi: 10.1371/journal.pone.0355679 (PMC13472385; doi:10.1371/journal.pone.0355679)
Supplement: Supplementary file 2 [file pone.0355679.s001.docx]

Inclusivity in global research

PLOS’ policy on inclusivity in global research aims to improve transparency in the reporting of research performed outside of researchers’ own country or community and ensures that PLOS publications reporting global research adhere to high standards for research ethics and authorship. Authors of relevant research articles may be asked to complete the questionnaire below, which outlines ethical, cultural, and scientific considerations specific to inclusivity in global research. This questionnaire may be requested when researchers have travelled to a different country to conduct research, if research uses samples collected in another country, research with Indigenous populations or their lands, or if research is on cultural artefacts. Researchers travelling to another country solely to use laboratory equipment will not normally be required to complete the questionnaire. However, the questionnaire can be requested at the journal’s discretion for any submission – if you have been requested to complete this questionnaire by the PLOS journal you submitted to, please do so.

Please complete the questionnaire below and include this as a Supporting Information file with your manuscript. Note that if your paper is accepted for publication, this checklist will be published with your article in the supporting information files. Please ensure that you reference the checklist in the main body of your manuscript. We suggest adding a subsection ‘Inclusivity in global research’ to your Methods section and adding the following sentence: “Additional information regarding the ethical, cultural, and scientific considerations specific to inclusivity in global research is included in the Supporting Information (SX Checklist)”

The questions have been designed to be applicable to a wide range of study types, and there are subsections for both human subjects research and non-human subjects research. If any of the questions are not relevant to your research please mark them as “N/A” as appropriate.

**Ethical considerations, permits and authorship**

*This section is applicable to all research types.*

Provide details as to who granted permissions and/or consent for the study to take place in the Methods section of your manuscript. This should include the names of **all** ethics boards, governmental organizations, community leaders or other bodies that provided approval for the study. If individuals provided approval refer to these people by their role or title but do not list their name(s).

Reported on page number: The Research Ethics Review Board (RERB) at the Simon Diedong Dombo University of Business and Integrated Development Studies (SDUBIDS), Ghana, granted ethical approval for the conduct of this research. Also, we have attached the ethical clearance certificate for your review. This can be found on page 8 of the manuscript.

If there were any deviations from the study protocol after approval was obtained please provide details of these changes in the Methods section of your manuscript.
Did this study involve local collaborators that are residents of the country where the research was conducted or members of the community studied? If you do not have any authors from said communities, please provide an explanation for this below.

All the authors are from the Upper West Region of Ghana, where this study was conducted. Stakeholders who participated in the research have been duely acknowledged in page 25 of the manuscript. Sincere thanks goes to the crop officers at the Department of Agriculture, the community leaders, and the data collectors who have contributed to this research.

Reported on page number: Not applicable

Everyone listed as an author should meet PLOS’ criteria for authorship and all individuals who meet these criteria should be included in the author byline, rather than the acknowledgements. For further information please see the journal’s Authorship Policy.

All authors meet the criteria. The authors declare no conflict of interest.

**Human subjects research (e.g. health research, medical research, cross-cultural psychology)**

Did you obtain written informed consent from a representative of the local community or region before the research took place? How did you establish who speaks for the community? Details of written informed consent obtained from study participants should be reported separately in the Methods section of your manuscript.

In our research conducted in the Upper West Region of Ghana, we engaged directly with smallholder farmers who practice climate-smart agriculture, farmer-based organizations (FBOs), Agric Extension Agents, and representatives of organizations that promote climate-smart agriculture. All of whom were adults, to obtain written informed consent before initiating the study. This engagement was crucial in ensuring the research was conducted ethically. We explained the purpose, methods, potential benefits, and any risks associated with the research to the participants before engaging them for their views. To establish who speaks for the FBOs and organizations, we undertook a thorough process, including preliminary visits, consultations with other members of staff, and open discussions with FBOs. This approach helped us to identify the right representatives to engage.

In the Methods section of our manuscript, we detailed the process of obtaining informed consent from the study participants (see page 8). We highlighted the steps taken to ensure that all participants were fully informed about the study's objectives, their right to withdraw at any time without being victimized, the measures taken to ensure confidentiality, and the benefits. We paid particular attention to cultural sensitivities, ensuring that the verbal consent and the communication language were understood by all participants. The consent process was verbal due to the large number of participants who were not literate. For those instances, verbal consent was obtained. By involving community representatives in the research process as enumerators, we maintained a high level of transparency and trust throughout the study. This inclusive approach ensured ethical integrity and significantly contributed to the smooth execution and success of our research in the Upper West Region of Ghana.

How did members of the local community provide input on the aims of the research investigation, its methodology, and its anticipated outcome(s)?

In our research, community members contributed to the study by volunteering as enumerators and respondents to the data collection instruments that were administered. This collaborative approach ensured that the research aims, methodology, and anticipated outcomes represented the true situation of the communities. Initial discussions helped refine the research questions to reflect community concerns, while feedback on methodology ensured culturally sensitive and ethically sound practices. Engaging the community in every step, using local languages and culturally respectful approaches, not only enhanced the relevance and impact of the research but also fostered a sense of ownership and partnership, crucial for the effective application of the findings.

When engaging with the local community, how did you ensure that the informed consent documents and other materials could be understood by local stakeholders?

We implemented a comprehensive approach to ensure that informed consent documents and other materials were comprehensible to local stakeholders in the Upper West Region of Ghana. All authors and data collectors are from the region, and language and cultural sensitivity were prioritized. Documents were translated into local languages and simplified to avoid misunderstanding. Cultural sensitivity was paramount, with all materials reviewed for cultural appropriateness. Community entry protocols were observed in the initial visits to the communities. Field assistants were effectively trained to help collect data.

Will the findings of the research be made available in an understandable format to stakeholders in the community where the study was conducted (e.g., via a presentation, summary report, copies of publications, etc.)? Please provide details of how this will be achieved.

To ensure the research findings benefit the stakeholders involved in the study, soft copies of the publications will be disseminated to individuals and on group pages on social media for easy accessibility. Other stakeholders may receive it via email, ensuring they have immediate access to the information to benefit a larger population. Farmer field schools and sensitization sessions will also serve as appropriate avenues to disseminate the findings of this research.

**Non-human subjects research using specimens/ animals collected as part of the study, or those housed in archival collections. Examples include archaeology, paleontology, botany and zoology.**

Did the permission you obtained from a local authority to perform the study include an agreement on access to outputs and benefit sharing? This may include procedures to enable fair distribution of the benefits and resources arising from the research performed. Please include any details of Prior Informed Consent and Benefit Sharing Agreements obtained. These may be required by field-specific regulations, for example the Convention on Biological Diversity (CBD) and the associated Nagoya Protocol.

N/A

If the material used in your study was imported, please A) provide the year it was imported and B) indicate whether permits were obtained to import/export the materials used, C) provide details of any permits obtained. If this information is not available, please indicate this.

N/A

If you used archival specimens, please state how the material used in your study was acquired by the institute it is held in, and provide details of any permits obtained for the original excavations/ sample collection. If this information is not available, please indicate this.

N/A

How was the potential cultural significance of the materials collected in your study to local communities considered in your research design? Were Indigenous peoples and/or local researchers and institutions involved with archaeological excavations / collection of specimens? If so, please provide a description of their involvement.

N/A

If your manuscript includes photographs of human remains please indicate whether authors obtained permission from descendants or affiliated cultural communities to do so.

N/A
