# Supplementary material for: Challenges to the adoption of climate-smart agricultural practices among smallholder farmers in semi-arid Ghana
Source: PLoS One. 2026 Aug 13;21(8):e0355679. doi: 10.1371/journal.pone.0355679 (PMC13472385; doi:10.1371/journal.pone.0355679)
Supplement: Supplementary file 4 [file pone.0355679.s004.docx]

**Response to Reviewers Comments**

**Challenges of Adoption of Climate-Smart Agricultural Practices Among Smallholder Farmers in Semi-Arid Ghana.**

| **Comment** | **Response** |
| --- | --- |
| **Reviewer1** |  |
| **Abstract** |  |
| The acronym AEAs is used without being defined. Please spell it out upon first mention. | **Response:** Thank you very much for your comment. AEO refers to Agricultural Extension officers and has been spelt out in full since it is used once in the abstract. |
| I recommend finishing the abstract with a sentence that highlights the policy relevance of your findings and how they can inform CSA programming and decision-making | **Response:** Besides demographic factors, the remaining factors are external and pose threats to farmers' capacity to adopt CSA. It is recommended that the government and Non-Governmental Organisations should support farmers with training, affordable farm inputs, and supportive policies to facilitate CSA adoption. |
| Introduction |  |
| The introduction is generally informative, but overly long and contains several instances of repeated information. I encourage substantial revision to improve conciseness, eliminate redundancy, and strengthen the narrative flow. | **Response:** We are grateful for this valuable comment. This has been revised accordingly. |
| Lines 43-44: The sentence is vague and should be rewritten for clarity. | **Response:** We appreciate this suggestion. The sentence has been recast as:  *Climate change is a significant global threat to development, with far-reaching implications for the agricultural sector.* |
| Line 55: The adverb “incredibly” is typically used in positive contexts. Since the text discusses negative climate impacts, please consider using a more appropriate alternative. | **Response:** Thank you very much. The text has been deleted. |
| Line 61: The acronym IPCC is not defined. Please provide the full term at first mention and specify which IPCC report you are referencing. | **Response:** We are grateful for this suggestion. The acronym has been defined at first use (see sentence below).  The sentence has been recast as follows:  The Working Group (WG) II contribution to the Sixth Assessment Report (AR6) of the Intergovernmental Panel on Climate Change (IPCC) predicts that climate change increases rural communities' exposure and vulnerability to a variety of pressures, including poverty, food insecurity, water scarcity, and land degradation due to their reliance on agriculture for livelihood (Pörtner et al., 20220) |
| Improving the structure and clarity of this section will greatly enhance the readability of the manuscript. | **Response:** We are really grateful for this suggestion. The manuscript has been revised accordingly. |
| Methodology |  |
| Lines 233-258: Consider summarizing the variables (demographic, motivational, and support-related factors) in a table. This will help readers to quickly understand the measurement framework and improve the clarity of the methods section. | **Response:** We appreciate this suggestion. We have clarified this in the revised manuscript. Please see Table 1. |
| Marital status categorization: why and how did you define these categories? | **Response:** Thank you for this clarification. These categories (Never Married = 0, Married = 1, Widowed/Divorced = 2) in marital studies have different family and livelihood needs that influence whether they adopt CSA or not. |
| Table 1: since the manuscript does not include any figures, a simple bar chart, presenting yes/no responses, would visually improve the presentation of the descriptive statistics more accessible. | **Response:** We appreciate the comment. This has been revised accordingly. The demographic characteristics have extracted and presented as graphs (See Figures 1A and B and 2A and B) |
| Coding of dependent variables: the binary coding using 1=yes and 2=no. Logistics regression conventionally requires 1 = event and 0 = non-event. Using 1 and 2 may affect the interpretation and should be corrected or clearly justified. | **Response:** Thank you very much for this comment. The coding has been reversed and the analysis re-run. |
| Sampling bias: the farmers were selected from communities already exposed to CSA interventions. This likely introduces selection bias, which could inflate the high CSA adoption rate. As a result, the findings may not be generalizable to communities without prior CSA intervention. Please acknowledge this limitation explicitly and discuss its implications. | **Response:** Although the farmers were selected from communities exposed to CSA practices, not every farmer in the community was exposed to CSA interventions. That notwithstanding, this limitation could introduce bias into the sample and inflate the CSA adoption rate. Thus, the study's findings cannot be generalized to include communities that have not been exposed to CSA interventions. |
| **Results and Discussion** |  |
| Please expand your interpretation of why married farmers were more likely to adopt CSA practices. | **Response:** The following paragraph has been included:  The likelihood that married smallholder farmers would adopt CSA practices may stem from ease of access to land and the security of land tenure, particularly in areas where land is owned by families, as in the study area. Atta-Aidoo and Antwi-Agyei (2025) explained that marriage serves as a means of accessing land in sub-Saharan Africa, given that families hold the majority of land. |
| The discussion includes some repetition and should be tightened to focus on the implications of the findings. | **Response:** Thank you very much for this insightful comment. The discussion has been reviewed to remove repetitions, and it has been tightened. |
| **Conclusion:** |  |
| The conclusion is generally clear but can be made more concise. Consider emphasizing the key insights, practical relevance, and limitations more succinctly. | **Response:** Thank you for the constructive feedback. The conclusion has been revised, and it now reads as:  *This study has highlighted the significant role of institutions in influencing CSA adoption through agricultural education and training programs using demonstration farms and farmer field schools. As a result, the study concluded that to achieve effective CSA adoption among smallholder farmers, existing institutions must be strengthened to implement relevant policies that support its adoption. It is crucial to foster synergies among the government, the private sector, and non-governmental organizations (NGOs) to establish an effective institutional framework that supports and promotes CSA in the agricultural sector. Similarly, supportive programmes and policy interventions should emphasize CSA adoption. To boost domestic productivity, policymakers should encourage and provide technical assistance, such as simple machinery, to smallholder farmers who adopt CSA practices that promote soil fertility and productivity. However, given that this research was time-limited and budget-constrained, it should be replicated across multiple locations to provide policymakers and practitioners with a clearer understanding of the factors influencing CSA adoption. Based on the findings, policymakers and non-governmental organizations (NGOs) that promote CSA practices should strengthen financial support systems and extension services for farmers, and provide more training on CSA. Relevant stakeholders should also consider the socio-demographic characteristics of smallholder farmers when promoting CSA.*  *In response to the challenges posed by global climate change, farming operations are now more successful due to CSA. A few studies have examined the factors affecting the level of CSA adoption among smallholder farmers in Semi-Arid Ghana. This study closes that gap by examining the factors influencing the extent to which smallholder farmers in the UWR of Ghana adopt climate-smart agricultural practices. Farmers in the study area generallypractice crop diversification, water conservation, the use of improved seeds, and soil fertility management (crop residues, animal manure, and compost). The study found that education, age, marital status, improvements in crop yields, reductions in input costs, support from extension officers, financial support, and education/training were factors associated with CSA adoption among farmers in the UWR.* |
| **Reviewer #2** |  |
| The manuscript is generally contributes to an important field, but substantial improvements in the problem framing, methodological description and discussion are necessary before it can be considered for publication. | **Response:** We appreciate this insight from the reviewers and have clarified it in the manuscript. |
| Overall Evaluation  The manuscript addresses an important and timely topic ‘Adoption of Climate-Smart Agricultural Practices among smallholder farmers in semi-arid Ghana’. The study is relevant for agricultural policy, adaptation planning, and rural development. The paper has potential, but it requires major revisions to improve clarity, methodological rigor, and the strength of the conclusions | **Response:** The method section have been revised accordingly. |
| The topic should be revised to better align with the manuscript, as the current content places substantial emphasis on challenges that are not clearly reflected in the title. | **Response:** The topic has been revised accordingly. Challenges of Adoption of Climate-Smart Agricultural Practices Among Smallholder Farmers in Semi-Arid Ghana. |
| Abstract: The abstract is well written; however, I recommend adding a brief recommendation at the end that is clearly linked to the study’s key findings. | **Response:** This has been addressed in Reviewer 1's comments. |
| Keywords, please see the document I highlighted. | **Response:** Climate-smart agriculture, Logistic regression, Odds ratio, Smallholder farmers |
| Background information is too much and try to focus on your main topic and I would recommend to reduce it. Please strengthen the justification by highlighting what is unknown about CSAP adoption in semi-arid Ghana and how this study contributes new insights | **Response:** Thank you very much for this comment. We have revised the introduction section. |
| Materials and methods: The description of the sampling procedure is insufficient. Please provide: Sampling frame and sample size determination technique. | **Response:** All study communities (Busa, Buka, Bugbelle, Loggu, Tuori, Chaangu, Chapuri, Tolibr, Sentu, Gozir, and Silbelle) received interventions from organizations that promote CSA, and this was one of the criteria for selecting these communities. Also, only respondents aged 18 or older were included in the study. The estimated number of farmers in this age category for the study area was 455,271. |
| Most of the tables are not presented in a standard or reader-friendly format. Please revise and rewrite the tables to improve clarity, consistency, and overall presentation. | **Response:** Tables have been formatted |
| The discussion should be more deeply connected to existing empirical literature. | **Response:** The discussion has been revised to reflect the comment |
| Some findings appear intuitive (e.g., extension contact increases adoption), but the authors should explain why these relationships occur in this specific context. | **Response:** Access to CSA training through contact with AEAs is one key determinant of CSA adoption practices among smallholder farmers. In Ghana, the MoFA deploys AEAs to communities to assist smallholder farmers in their farming practices. The government uses them as a conduit to disseminate information, render training on new technologies, and distribute farm inputs such as fertilizers and improved seeds. These trainings provide farmers with the knowledge and skills to implement CSA practices. According to Nkuba et al. (2020), training plays a crucial role in exposing farmers to the supportive institutions, input sources, and credit facilities necessary for implementing CSA. This is consistent with the findings of Abegunde et al. (2020), Luu (2020), and Ndung’u et al. (2023), who observed that exposure through contact with AEAs has a statistically significant positive effect on CSA adoption in South Africa. Agricultural extension services have been proven to build farmers’ agricultural knowledge and skills, disseminate new technologies, and change farmers’ attitudes toward evolving farming practices (Zafarullah Khan et al., 2012). |
| Provide more context-specific interpretation relating to the characteristics of semi-arid Ghana | **Response:** Thank you very much for this comment. This has been incorporated into the study context. |

We would like to thank the reviewers for reviewing our paper and for providing constructive suggestions that have helped to improve the paper significantly. We are grateful.
