# Supplementary material for: Challenges to the adoption of climate-smart agricultural practices among smallholder farmers in semi-arid Ghana
Source: PLoS One. 2026 Aug 13;21(8):e0355679. doi: 10.1371/journal.pone.0355679 (PMC13472385; doi:10.1371/journal.pone.0355679)
Supplement: Supplementary file 5 [file pone.0355679.s002.docx]

Reviewer comment

Overall Evaluation

The manuscript addresses an important and timely topic ‘Adoption of Climate-Smart Agricultural Practices among smallholder farmers in semi-arid Ghana’. The study is relevant for agricultural policy, adaptation planning, and rural development. The paper has potential, but it requires major revisions to improve clarity, methodological rigor, and the strength of the conclusions

Detail Evaluation

1. The topic should be revised to better align with the manuscript, as the current content places substantial emphasis on challenges that are not clearly reflected in the title
2. Abstract: The abstract is well written; however, I recommend adding a brief recommendation at the end that is clearly linked to the study’s key findings.
3. Keywords, please see the document I highlighted.
4. Background information is too much and try to focus on your main topic and I would recommend to reduce it. Please strengthen the justification by highlighting what is unknown about CSAP adoption in semi-arid Ghana and how this study contributes new insights
5. Materials and methods: The description of the sampling procedure is insufficient. Please provide: Sampling frame and sample size determination technique.
6. Most of the tables are not presented in a standard or reader-friendly format. Please revise and rewrite the tables to improve clarity, consistency, and overall presentation.
7. The discussion should be more deeply connected to existing empirical literature.
8. Some findings appear intuitive (e.g., extension contact increases adoption), but the authors should explain why these relationships occur in this specific context.
9. Provide more context-specific interpretation relating to the characteristics of semi-arid Ghana

**Major Revision Required**

The manuscript contributes to an important field, but substantial improvements in problem framing, methodological description, and discussion are necessary before it can be considered for publication.
