# Supplementary material for: Challenges to the adoption of climate-smart agricultural practices among smallholder farmers in semi-arid Ghana
Source: PLoS One. 2026 Aug 13;21(8):e0355679. doi: 10.1371/journal.pone.0355679 (PMC13472385; doi:10.1371/journal.pone.0355679)
Supplement: Supplementary file 6 [file pone.0355679.s005.docx]

**Response to Reviewers Comments**

**Challenges to Adoption of Climate-Smart Agricultural Practices Among Smallholder Farmers in Semi-Arid Ghana.**

**Abstract**

**Comment**: The additional information on age, gender, education, and marital status is not fully supported by the analysis presented. For example, statements such as "men dominate the adoption of CSA practices" and "married farmers are more likely to adopt" are based on the proportion of respondents in the sample rather than the regression results. Since gender was not statistically significant, these statements should either be removed or revised to reflect the actual findings. In addition, the statements about younger farmers adopting more are inconsistent with the regression results and should be checked carefully. Also, you used some references in the abstract, but I think using references in the abstract section is not allowed.

**Response:** Thank you very much for this comment. We have revised the manuscript to include the regression results on the effect of age and education on CSA adoption. The section now reads;

*Demographic factors also shaped the adoption of CSA practices. Farmers within the ages of 26-45 were less likely to adopt CSA practices (OR = 0.050; p<0. 05), while farmers with formal education were more likely to adopt CSA practices (OR = 9.262; p<0. 00) after controlling for motivational and support system variables.*

Also, we have removed all the references in the abstract.

**Consistency of results**

**Comment**: There appear to be inconsistencies between the regression tables, the Results section, and the Abstract, particularly regarding the effect of age on CSA adoption. These interpretations should be checked carefully to ensure that they match the reported odds ratios.

**Response:** Inconsistent interpretations between regression tables, results section, and abstract have been resolved to match the reported odds ratios. The effect of age on CSA adoption is less likely. The result section now reads:

*However, while no significant association was found between age and CSA in Model 1, in Model 2, farmers between the ages of 26-35 (OR=0.102; p<0.1) were less likely to adopt CSA practices.*

Thank you very much for your constructive comments.
