# Supplementary material for: Challenges to the adoption of climate-smart agricultural practices among smallholder farmers in semi-arid Ghana
Source: PLoS One. 2026 Aug 13;21(8):e0355679. doi: 10.1371/journal.pone.0355679 (PMC13472385; doi:10.1371/journal.pone.0355679)
Supplement: Peer review history

# Peer review: Decision Letter 0

**DOI:** 10.1371/journal.pone.0355679.r001

**Authors:** Babak Ghazi

17 Nov 2025

Dear Dr. Molle,

Thank you for submitting your manuscript to PLOS ONE. After careful consideration, we feel that it has merit but does not fully meet PLOS ONE’s publication criteria as it currently stands. Therefore, we invite you to submit a revised version of the manuscript that addresses the points raised during the review process.

==============================

Both reviewers agree that the manuscript addresses an important topic but requires some improvements in methodology, organization, and presentation. You need to revise your manuscript to improve the method section, strengthen the discussion, and enhance the overall coherence.

=============================

Please submit your revised manuscript by Jan 01 2026 11:59PM. If you will need more time than this to complete your revisions, please reply to this message or contact the journal office at plosone@plos.org. When you're ready to submit your revision, log on to https://www.editorialmanager.com/pone/ and select the 'Submissions Needing Revision' folder to locate your manuscript file.

- A rebuttal letter that responds to each point raised by the academic editor and reviewer(s). You should upload this letter as a separate file labeled 'Response to Reviewers'.
- A marked-up copy of your manuscript that highlights changes made to the original version. You should upload this as a separate file labeled 'Revised Manuscript with Track Changes'.
- An unmarked version of your revised paper without tracked changes. You should upload this as a separate file labeled 'Manuscript'.

If you would like to make changes to your financial disclosure, please include your updated statement in your cover letter. Guidelines for resubmitting your figure files are available below the reviewer comments at the end of this letter.

If applicable, we recommend that you deposit your laboratory protocols in protocols.io to enhance the reproducibility of your results. Protocols.io assigns your protocol its own identifier (DOI) so that it can be cited independently in the future. For instructions see: https://journals.plos.org/plosone/s/submission-guidelines#loc-laboratory-protocols. Additionally, PLOS ONE offers an option for publishing peer-reviewed Lab Protocol articles, which describe protocols hosted on protocols.io. Read more information on sharing protocols at https://plos.org/protocols?utm_medium=editorial-email&utm_source=authorletters&utm_campaign=protocols.

We look forward to receiving your revised manuscript.

Kind regards,

Babak Ghazi, PhD

Academic Editor

PLOS ONE

Journal Requirements:

When submitting your revision, we need you to address these additional requirements.

1. Please ensure that your manuscript meets PLOS ONE's style requirements, including those for file naming. The PLOS ONE style templates can be found at

https://journals.plos.org/plosone/s/file?id=wjVg/PLOSOne_formatting_sample_main_body.pdf and

https://journals.plos.org/plosone/s/file?id=ba62/PLOSOne_formatting_sample_title_authors_affiliations.pdf

2. Please include a complete copy of PLOS’ questionnaire on inclusivity in global research in your revised manuscript. Our policy for research in this area aims to improve transparency in the reporting of research performed outside of researchers’ own country or community. The policy applies to researchers who have travelled to a different country to conduct research, research with Indigenous populations or their lands, and research on cultural artefacts. The questionnaire can also be requested at the journal’s discretion for any other submissions, even if these conditions are not met.  Please find more information on the policy and a link to download a blank copy of the questionnaire here: https://journals.plos.org/plosone/s/best-practices-in-research-reporting. Please upload a completed version of your questionnaire as Supporting Information when you resubmit your manuscript.

3. When completing the data availability statement of the submission form, you indicated that you will make your data available on acceptance. We strongly recommend all authors decide on a data sharing plan before acceptance, as the process can be lengthy and hold up publication timelines. Please note that, though access restrictions are acceptable now, your entire data will need to be made freely accessible if your manuscript is accepted for publication. This policy applies to all data except where public deposition would breach compliance with the protocol approved by your research ethics board. If you are unable to adhere to our open data policy, please kindly revise your statement to explain your reasoning and we will seek the editor's input on an exemption. Please be assured that, once you have provided your new statement, the assessment of your exemption will not hold up the peer review process.

4. Please amend the manuscript submission data (via Edit Submission) to include author Suleman Ansumah Saaka

5. Please amend your authorship list in your manuscript file to include author Sulemana Ansumah Saaka

6. We note that Figure 1 in your submission contain map images which may be copyrighted. All PLOS content is published under the Creative Commons Attribution License (CC BY 4.0), which means that the manuscript, images, and Supporting Information files will be freely available online, and any third party is permitted to access, download, copy, distribute, and use these materials in any way, even commercially, with proper attribution. For these reasons, we cannot publish previously copyrighted maps or satellite images created using proprietary data, such as Google software (Google Maps, Street View, and Earth). For more information, see our copyright guidelines: http://journals.plos.org/plosone/s/licenses-and-copyright.

We require you to either (a) present written permission from the copyright holder to publish these figures specifically under the CC BY 4.0 license, or (b) remove the figures from your submission:

a. You may seek permission from the original copyright holder of Figure 1 to publish the content specifically under the CC BY 4.0 license.

We recommend that you contact the original copyright holder with the Content Permission Form (http://journals.plos.org/plosone/s/file?id=7c09/content-permission-form.pdf) and the following text:

“I request permission for the open-access journal PLOS ONE to publish XXX under the Creative Commons Attribution License (CCAL) CC BY 4.0 (http://creativecommons.org/licenses/by/4.0/). Please be aware that this license allows unrestricted use and distribution, even commercially, by third parties. Please reply and provide explicit written permission to publish XXX under a CC BY license and complete the attached form.”

Please upload the completed Content Permission Form or other proof of granted permissions as an ""Other"" file with your submission.

In the figure caption of the copyrighted figure, please include the following text: “Reprinted from [ref] under a CC BY license, with permission from [name of publisher], original copyright [original copyright year].”

b. If you are unable to obtain permission from the original copyright holder to publish these figures under the CC BY 4.0 license or if the copyright holder’s requirements are incompatible with the CC BY 4.0 license, please either i) remove the figure or ii) supply a replacement figure that complies with the CC BY 4.0 license. Please check copyright information on all replacement figures and update the figure caption with source information. If applicable, please specify in the figure caption text when a figure is similar but not identical to the original image and is therefore for illustrative purposes only.

The following resources for replacing copyrighted map figures may be helpful:

USGS National Map Viewer (public domain): http://viewer.nationalmap.gov/viewer/

The Gateway to Astronaut Photography of Earth (public domain): http://eol.jsc.nasa.gov/sseop/clickmap/

Maps at the CIA (public domain): https://www.cia.gov/library/publications/the-world-factbook/index.html and https://www.cia.gov/library/publications/cia-maps-publications/index.html

NASA Earth Observatory (public domain): http://earthobservatory.nasa.gov/

Landsat: http://landsat.visibleearth.nasa.gov/

USGS EROS (Earth Resources Observatory and Science (EROS) Center) (public domain): http://eros.usgs.gov/#

Natural Earth (public domain): http://www.naturalearthdata.com/

7. Please include captions for your Supporting Information files at the end of your manuscript, and update any in-text citations to match accordingly. Please see our Supporting Information guidelines for more information: http://journals.plos.org/plosone/s/supporting-information.

8. If the reviewer comments include a recommendation to cite specific previously published works, please review and evaluate these publications to determine whether they are relevant and should be cited. There is no requirement to cite these works unless the editor has indicated otherwise.

[Note: HTML markup is below. Please do not edit.]

Reviewers' comments:

Reviewer's Responses to Questions

**Comments to the Author**

1. Is the manuscript technically sound, and do the data support the conclusions?

Reviewer #1: Partly

Reviewer #2: Yes

**********

2. Has the statistical analysis been performed appropriately and rigorously?

Reviewer #1: Yes

Reviewer #2: Yes

**********

3. Have the authors made all data underlying the findings in their manuscript fully available?

The PLOS Data policy

Reviewer #1: Yes

Reviewer #2: Yes

**********

4. Is the manuscript presented in an intelligible fashion and written in standard English?

Reviewer #1: Yes

Reviewer #2: No

**********

Reviewer #1: It was my pleasure to review the paper entitled “Adoption of Climate-Smart Agricultural Practices Among Smallholder Farmers in Semi-Arid Ghana”. Despite the importance of climate smart agriculture practices in vulnerable areas like Ghana and assessing the influence of different factors on farmers’ decision making, the manuscript requires major revision before it can be considered for publication. Below are my comments:

Title: Good

Abstract:

1. The acronym AEAs is used without being defined. Please spell it out upon first mention.

2. I recommend finishing the abstract with a sentence that highlights the policy relevance of your findings and how they can inform CSA programming and decision-making.

Introduction:

The Introduction is generally informative but overly long and contains several instances of repeated information. I encourage substantial revision to improve conciseness, eliminate redundancy, and strengthen the narrative flow. I also have some other comments include:

1. Lines 43–44: The sentence is vague and should be rewritten for clarity.

2. Line 55: The adverb “incredibly” is typically used in positive contexts. Since the text discusses negative climate impacts, please consider using a more appropriate alternative.

3. Line 61: The acronym IPCC is not defined. Please provide the full term at first mention and specify which IPCC report you are referencing.

Improving the structure and clarity of this section will greatly enhance the readability of the manuscript.

Methodology:

1. Lines 233–258: Consider summarizing the variables (demographic, motivational, and support-related factors) in a table. This will help readers quickly understand the measurement framework and improve the clarity of the Methods section.

2. Marital status categorization: why and how did you defined these categories?

3. Table 1: Since the manuscript does not include any figures, a simple bar chart presenting yes/no responses would visually improve the presentation and make the descriptive findings more accessible.

4. Coding of the dependent variable: The binary coding uses 1 = Yes and 2 = No. Logistic regression conventionally requires 1 = event and 0 = non-event. Using 1 and 2 may affect interpretation and should be corrected or clearly justified.

5. Sampling bias: Farmers were selected from communities already exposed to CSA interventions. This likely introduces selection bias, which could inflate the high CSA adoption rate. As a result, the findings may not be generalizable to communities without prior CSA intervention. Please acknowledge this limitation explicitly and discuss its implications.

Results and Discussion

1. Please expand your interpretation of why married farmers were more likely to adopt CSA practices.

2. The Discussion includes some repetition and should be tightened to focus more clearly on the implications of the findings.

Conclusion:

The conclusion is generally clear but can be made more concise. Consider emphasizing the key insights, practical relevance, and limitations more succinctly.

Reviewer #2: The manuscript contributes to an important field, but substantial improvements in problem framing, methodological description, and discussion are necessary before it can be considered for publication.

Please see the attached documents

**********

what does this mean?). If published, this will include your full peer review and any attached files.

If you choose “no”, your identity will remain anonymous but your review may still be made public.

**Do you want your identity to be public for this peer review?**  For information about this choice, including consent withdrawal, please see our Privacy Policy

Reviewer #1: No

Reviewer #2: No

**********

[NOTE: If reviewer comments were submitted as an attachment file, they will be attached to this email and accessible via the submission site. Please log into your account, locate the manuscript record, and check for the action link "View Attachments". If this link does not appear, there are no attachment files.]

To ensure your figures meet our technical requirements, please review our figure guidelines: https://journals.plos.org/plosone/s/figures

You may also use PLOS’s free figure tool, NAAS, to help you prepare publication quality figures: https://journals.plos.org/plosone/s/figures#loc-tools-for-figure-preparation.

NAAS will assess whether your figures meet our technical requirements by comparing each figure against our figure specifications.

Attachment Submitted filename: Reviewer comment.docx

Attachment Submitted filename: PONE-D-25-35144 (1).pdf

# Author comment: Author response to Decision Letter 1

**DOI:** 10.1371/journal.pone.0355679.r002

19 Feb 2026

Comment Response

Reviewer1

Abstract

The acronym AEAs is used without being defined. Please spell it out upon first mention. Response: Thank you very much for your comment. AEO refers to Agricultural Extension officers and has been spelt out in full since it is used once in the abstract.

I recommend finishing the abstract with a sentence that highlights the policy relevance of your findings and how they can inform CSA programming and decision-making Response: Besides demographic factors, the remaining factors are external and pose threats to farmers' capacity to adopt CSA. It is recommended that the government and Non-Governmental Organisations should support farmers with training, affordable farm inputs, and supportive policies to facilitate CSA adoption.

Introduction

The introduction is generally informative, but overly long and contains several instances of repeated information. I encourage substantial revision to improve conciseness, eliminate redundancy, and strengthen the narrative flow. Response: We are grateful for this valuable comment. This has been revised accordingly.

Lines 43-44: The sentence is vague and should be rewritten for clarity. Response: We appreciate this suggestion. The sentence has been recast as:

Climate change is a significant global threat to development, with far-reaching implications for the agricultural sector.

Line 55: The adverb “incredibly” is typically used in positive contexts. Since the text discusses negative climate impacts, please consider using a more appropriate alternative. Response: Thank you very much. The text has been deleted.

Line 61: The acronym IPCC is not defined. Please provide the full term at first mention and specify which IPCC report you are referencing. Response: We are grateful for this suggestion. The acronym has been defined at first use (see sentence below).

The sentence has been recast as follows:

The Working Group (WG) II contribution to the Sixth Assessment Report (AR6) of the Intergovernmental Panel on Climate Change (IPCC) predicts that climate change increases rural communities' exposure and vulnerability to a variety of pressures, including poverty, food insecurity, water scarcity, and land degradation due to their reliance on agriculture for livelihood (Pörtner et al., 20220)

Improving the structure and clarity of this section will greatly enhance the readability of the manuscript. Response: We are really grateful for this suggestion. The manuscript has been revised accordingly.

Methodology

Lines 233-258: Consider summarizing the variables (demographic, motivational, and support-related factors) in a table. This will help readers to quickly understand the measurement framework and improve the clarity of the methods section. Response: We appreciate this suggestion. We have clarified this in the revised manuscript. Please see Table 1.

Marital status categorization: why and how did you define these categories? Response: Thank you for this clarification. These categories (Never Married = 0, Married = 1, Widowed/Divorced = 2) in marital studies have different family and livelihood needs that influence whether they adopt CSA or not.

Table 1: since the manuscript does not include any figures, a simple bar chart, presenting yes/no responses, would visually improve the presentation of the descriptive statistics more accessible. Response: We appreciate the comment. This has been revised accordingly. The demographic characteristics have extracted and presented as graphs (See Figures 1A and B and 2A and B)

Coding of dependent variables: the binary coding using 1=yes and 2=no. Logistics regression conventionally requires 1 = event and 0 = non-event. Using 1 and 2 may affect the interpretation and should be corrected or clearly justified. Response: Thank you very much for this comment. The coding has been reversed and the analysis re-run.

Sampling bias: the farmers were selected from communities already exposed to CSA interventions. This likely introduces selection bias, which could inflate the high CSA adoption rate. As a result, the findings may not be generalizable to communities without prior CSA intervention. Please acknowledge this limitation explicitly and discuss its implications. Response: Although the farmers were selected from communities exposed to CSA practices, not every farmer in the community was exposed to CSA interventions. That notwithstanding, this limitation could introduce bias into the sample and inflate the CSA adoption rate. Thus, the study's findings cannot be generalized to include communities that have not been exposed to CSA interventions.

Results and Discussion

Please expand your interpretation of why married farmers were more likely to adopt CSA practices. Response: The following paragraph has been included:

The likelihood that married smallholder farmers would adopt CSA practices may stem from ease of access to land and the security of land tenure, particularly in areas where land is owned by families, as in the study area. Atta-Aidoo and Antwi-Agyei (2025) explained that marriage serves as a means of accessing land in sub-Saharan Africa, given that families hold the majority of land.

The discussion includes some repetition and should be tightened to focus on the implications of the findings. Response: Thank you very much for this insightful comment. The discussion has been reviewed to remove repetitions, and it has been tightened.

Conclusion:

The conclusion is generally clear but can be made more concise. Consider emphasizing the key insights, practical relevance, and limitations more succinctly. Response: Thank you for the constructive feedback. The conclusion has been revised, and it now reads as:

This study has highlighted the significant role of institutions in influencing CSA adoption through agricultural education and training programs using demonstration farms and farmer field schools. As a result, the study concluded that to achieve effective CSA adoption among smallholder farmers, existing institutions must be strengthened to implement relevant policies that support its adoption. It is crucial to foster synergies among the government, the private sector, and non-governmental organizations (NGOs) to establish an effective institutional framework that supports and promotes CSA in the agricultural sector. Similarly, supportive programmes and policy interventions should emphasize CSA adoption. To boost domestic productivity, policymakers should encourage and provide technical assistance, such as simple machinery, to smallholder farmers who adopt CSA practices that promote soil fertility and productivity. However, given that this research was time-limited and budget-constrained, it should be replicated across multiple locations to provide policymakers and practitioners with a clearer understanding of the factors influencing CSA adoption. Based on the findings, policymakers and non-governmental organizations (NGOs) that promote CSA practices should strengthen financial support systems and extension services for farmers, and provide more training on CSA. Relevant stakeholders should also consider the socio-demographic characteristics of smallholder farmers when promoting CSA.

In response to the challenges posed by global climate change, farming operations are now more successful due to CSA. A few studies have examined the factors affecting the level of CSA adoption among smallholder farmers in Semi-Arid Ghana. This study closes that gap by examining the factors influencing the extent to which smallholder farmers in the UWR of Ghana adopt climate-smart agricultural practices. Farmers in the study area generallypractice crop diversification, water conservation, the use of improved seeds, and soil fertility management (crop residues, animal manure, and compost). The study found that education, age, marital status, improvements in crop yields, reductions in input costs, support from extension officers, financial support, and education/training were factors associated with CSA adoption among farmers in the UWR.

Reviewer #2

The manuscript is generally contributes to an important field, but substantial improvements in the problem framing, methodological description and discussion are necessary before it can be considered for publication. Response: We appreciate this insight from the reviewers and have clarified it in the manuscript.

Overall Evaluation

The manuscript addresses an important and timely topic ‘Adoption of Climate-Smart Agricultural Practices among smallholder farmers in semi-arid Ghana’. The study is relevant for agricultural policy, adaptation planning, and rural development. The paper has potential, but it requires major revisions to improve clarity, methodological rigor, and the strength of the conclusions Response: The method section have been revised accordingly.

The topic should be revised to better align with the manuscript, as the current content places substantial emphasis on challenges that are not clearly reflected in the title.

Response: The topic has been revised accordingly. Challenges of Adoption of Climate-Smart Agricultural Practices Among Smallholder Farmers in Semi-Arid Ghana.

Abstract: The abstract is well written; however, I recommend adding a brief recommendation at the end that is clearly linked to the study’s key findings. Response: This has been addressed in Reviewer 1's comments.

Keywords, please see the document I highlighted. Response: Climate-smart agriculture, Logistic regression, Odds ratio, Smallholder farmers

Background information is too much and try to focus on your main topic and I would recommend to reduce it. Please strengthen the justification by highlighting what is unknown about CSAP adoption in semi-arid Ghana and how this study contributes new insights Response: Thank you very much for this comment. We have revised the introduction section.

Materials and methods: The description of the sampling procedure is insufficient. Please provide: Sampling frame and sample size determination technique. Response: All study communities (Busa, Buka, Bugbelle, Loggu, Tuori, Chaangu, Chapuri, Tolibr, Sentu, Gozir, and Silbelle) received interventions from organizations that promote CSA, and this was one of the criteria for selecting these communities. Also, only respondents aged 18 or older were included in the study. The estimated number of farmers in this age category for the study area was 455,271.

Most of the tables are not presented in a standard or reader-friendly format. Please revise and rewrite the tables to improve clarity, consistency, and overall presentation. Response: Tables have been formatted

The discussion should be more deeply connected to existing empirical literature. Response: The discussion has been revised to reflect the comment

Some findings appear intuitive (e.g., extension contact increases adoption), but the authors should explain why these relationships occur in this specific context. Response: Access to CSA training through contact with AEAs is one key determinant of CSA adoption practices among smallholder farmers. In Ghana, the MoFA deploys AEAs to communities to assist smallholder farmers in their farming practices. The government uses them as a conduit to disseminate information, render training on new technologies, and distribute farm inputs such as fertilizers and improved seeds. These trainings provide farmers with the knowledge and skills to implement CSA practices. According to Nkuba et al. (2020), training plays a crucial role in exposing farmers to the supportive institutions, input sources, and credit facilities necessary for implementing CSA. This is consistent with the findings of Abegunde et al. (2020), Luu (2020), and Ndung’u et al. (2023), who observed that exposure through contact with AEAs has a statistically significant positive effect on CSA adoption in South Africa. Agricultural extension services have been proven to build farmers’ agricultural knowledge and skills, disseminate new technologies, and change farmers’ attitudes toward evolving farming practices (Zafarullah Khan et al., 2012).

Provide more context-specific interpretation relating to the characteristics of semi-arid Ghana Response: Thank you very much for this comment. This has been incorporated into the study context.

Attachment Submitted filename: Response to Reviewers.docx

# Peer review: Decision Letter 1

**DOI:** 10.1371/journal.pone.0355679.r003

**Authors:** Babak Ghazi

28 Apr 2026

PLOS One

Dear Dr. Molle,

Thank you for submitting your manuscript to PLOS ONE. After careful consideration, we feel that it has merit but does not fully meet PLOS ONE’s publication criteria as it currently stands. Therefore, we invite you to submit a revised version of the manuscript that addresses the points raised during the review process.

==============================

Thank you for submitting the revised manuscript. Although the manuscript has improved, the reviewer noted that some concerns remain insufficiently addressed, particularly regarding clarity in the abstract, conciseness and focus of the introduction, the study’s novelty compared with previous work, and the implications of the sample distribution for the robustness and generalizability of the findings.

Please revise the manuscript carefully and provide a detailed response to the reviewer comments, explaining how each has been addressed. Please make sure you address all comments from the reviewer(s) carefully to avoid an additional round of review.

==============================

Please submit your revised manuscript by Jun 12 2026 11:59PM. If you will need more time than this to complete your revisions, please reply to this message or contact the journal office at plosone@plos.org. When you're ready to submit your revision, log on to https://www.editorialmanager.com/pone/ and select the 'Submissions Needing Revision' folder to locate your manuscript file.

Please include the following items when submitting your revised manuscript:

- A letter that responds to each point raised by the academic editor and reviewer(s). You should upload this letter as a separate file labeled 'Response to Reviewers'.
- A marked-up copy of your manuscript that highlights changes made to the original version. You should upload this as a separate file labeled 'Revised Manuscript with Track Changes'.
- An unmarked version of your revised paper without tracked changes. You should upload this as a separate file labeled 'Manuscript'.

If you would like to make changes to your financial disclosure, please include your updated statement in your cover letter. Guidelines for resubmitting your figure files are available below the reviewer comments at the end of this letter.

If applicable, we recommend that you deposit your laboratory protocols in protocols.io to enhance the reproducibility of your results. Protocols.io assigns your protocol its own identifier (DOI) so that it can be cited independently in the future. For instructions see: https://journals.plos.org/plosone/s/submission-guidelines#loc-laboratory-protocols. Additionally, PLOS ONE offers an option for publishing peer-reviewed Lab Protocol articles, which describe protocols hosted on protocols.io. Read more information on sharing protocols at https://plos.org/protocols?utm_medium=editorial-email&utm_source=authorletters&utm_campaign=protocols.

As the corresponding author, your ORCID iD is verified in the submission system and will appear in the published article. PLOS supports the use of ORCID, and we encourage all coauthors to register for an ORCID iD and use it as well. Please encourage your coauthors to verify their ORCID iD within the submission system before final acceptance, as unverified ORCID iDs will not appear in the published article. *Only*  the individual author can complete the verification step; PLOS staff *cannot*  verify ORCID iDs on behalf of authors.

We look forward to receiving your revised manuscript.

Kind regards,

Babak Ghazi, PhD

Academic Editor

PLOS One

Journal Requirements:

If the reviewer comments include a recommendation to cite specific previously published works, please review and evaluate these publications to determine whether they are relevant and should be cited. There is no requirement to cite these works unless the editor has indicated otherwise.

[Note: HTML markup is below. Please do not edit.]

Reviewers' comments:

Reviewer's Responses to Questions

**Comments to the Author**

Reviewer #1: (No Response)

**********

2. Is the manuscript technically sound, and do the data support the conclusions?

Reviewer #1: Partly

**********

3. Has the statistical analysis been performed appropriately and rigorously?

Reviewer #1: (No Response)

**********

4. Have the authors made all data underlying the findings in their manuscript fully available?

The PLOS Data policy

Reviewer #1: (No Response)

**********

5. Is the manuscript presented in an intelligible fashion and written in standard English?

Reviewer #1: (No Response)

**********

Reviewer #1: Thank you for modifying the manuscript and addressing the comments. Below are my comments that need to be addressed before proceeding:

Minor issues:

In Abstract:

1) The abbreviation “AEA” still needs to be defined at its first occurrence.

2) The sentence “Moreover, the study indicated a significant relationship between demographic factors such as age, education, and marital status of farmers and their decision to adopt CSA practices” could be more informative by specifying which categories showed higher adoption (e.g., younger farmers?, more educated farmers?, married farmers?, etc.).

In Introduction:

3) This section still appears too wordy and would benefit from further editing to make it shorter and more focused.

Major issues:

In Introduction:

1) What differentiates this study from previous studies that reported similar findings? For instance, many studies worldwide have shown that factors such as education level and age influence farmers’ decision-making. What specifically makes this study distinct from other similar studies? This should be explicitly clarified in the introduction.

In Results:

2) Figure 1 shows a highly skewed sample with large differences in the number of respondents across categories. For example, around 60% of the participants belong to the 26–35 age group, while the other categories are underrepresented. How might this imbalance bias the findings?

A similar issue exists for marital status, where around 85% of participants are married. Please discuss how this imbalance might influence the results.

Such skewed distributions may lead to unstable estimates for minority groups and cause the regression results to primarily reflect the dominant categories, like what the current results show. The authors should discuss how these imbalances may have influenced the robustness and generalizability of the findings.

**********

what does this mean?). If published, this will include your full peer review and any attached files.

If you choose “no”, your identity will remain anonymous but your review may still be made public.

**Do you want your identity to be public for this peer review?**  For information about this choice, including consent withdrawal, please see our Privacy Policy

Reviewer #1: No

**********

[NOTE: If reviewer comments were submitted as an attachment file, they will be attached to this email and accessible via the submission site. Please log into your account, locate the manuscript record, and check for the action link "View Attachments". If this link does not appear, there are no attachment files.]

To ensure your figures meet our technical requirements, please review our figure guidelines: https://journals.plos.org/plosone/s/figures

You may also use PLOS’s free figure tool, NAAS, to help you prepare publication quality figures: https://journals.plos.org/plosone/s/figures#loc-tools-for-figure-preparation.

NAAS will assess whether your figures meet our technical requirements by comparing each figure against our figure specifications.

# Author comment: Author response to Decision Letter 2

**DOI:** 10.1371/journal.pone.0355679.r004

1 Jul 2026

Challenges to Adoption of Climate-Smart Agricultural Practices Among Smallholder Farmers in Semi-Arid Ghana.

In Abstract:

Comment: The abbreviation “AEA” still needs to be defined at its first occurrence.

Response: AEA has been spelt out in full since it is used once in the abstract

Comment: The sentence “Moreover, the study indicated a significant relationship between demographic factors such as age, education, and marital status of farmers and their decision to adopt CSA practices” could be more informative by specifying which categories showed higher adoption (e.g., younger farmers? more educated farmers? married farmers? etc.).

Response: This has been specified as follows:

For gender: 69.5% male, 30.5% female respectively adopt CSA practices. Indicating that men dominate the adoption of CSA practices. Even though, women’s participation is rising (Van Nieuwkoop et al., 2022).

Married farmers are more likely to adopt CSA practices as 85% of respondents were married. This is not surprising as most communities in the study area engage in family-based farming.

Regarding age, 91.25% youth, 8.75% aged was revealed. Youth drive CSA adoption; while older farmers are less likely. This was backed by a study by Gazali & Awudu, 2019.

Majority had basic/secondary education – education enhances awareness, openness to innovation, and adoption of CSA practices.

In Introduction:

Comment: This section still appears too wordy and would benefit from further editing to make it shorter and more focused.

Response: This has been done.

In Introduction:

Comment: What differentiates this study from previous studies that reported similar findings? For instance, many studies worldwide have shown that factors such as education level and age influence farmers’ decision-making. What specifically makes this study distinct from other similar studies? This should be explicitly clarified in the introduction.

Response: Other variables considered in the study, such as agricultural extension, input costs, training or education on CSA practices, government policies, and financial incentives, provided a clearer picture of the challenges to adopting CSA practices, distinguishing this study from previous studies that reported similar findings.

In Results:

Comment: Figure 1 shows a highly skewed sample with large differences in the number of respondents across categories. For example, around 60% of the participants belong to the 26–35 age group, while the other categories are underrepresented. How might this imbalance bias the findings?

A similar issue exists for marital status, where around 85% of participants are married. Please discuss how this imbalance might influence the results.

Such skewed distributions may lead to unstable estimates for minority groups and cause the regression results to primarily reflect the dominant categories, like what the current results show. The authors should discuss how these imbalances may have influenced the robustness and generalizability of the findings.

Response: The skewness of respondents' ages toward the 26-35 age group could be attributed to favourable government policies, such as the Planting and Rearing for Food and Jobs policy, which attracted many young people to farming. Although the dataset exhibited significant skewness, the conclusions were robust to alternative analytical approaches. Specifically, alternative statistical tests that were run equally produced similar results. Due to the skewness, we used semi-parametric tests (like robust standard errors), but parametric tests (like t-tests and ordinary least squares) were also used for robustness checks, and they yielded similar conclusions given the large sample size.

In the case of marital status, the skewness is attributed to farming being the primary source of livelihood for married families in rural Ghana, where the study was conducted.

Contextually, the skewness itself is a characteristic of the population, not just the study sample (especially given that the study setting is typically patriarchal).

Despite the skew, the sample was representative of the target population because simple random sampling was used, supporting generalizability.

# Peer review: Decision Letter 2

**DOI:** 10.1371/journal.pone.0355679.r005

**Authors:** Babak Ghazi

5 Jul 2026

Dear Dr. Molle,

Thank you for submitting your manuscript to PLOS ONE. After careful consideration, we feel that it has merit but does not fully meet PLOS ONE’s publication criteria as it currently stands. Therefore, we invite you to submit a revised version of the manuscript that addresses the points raised during the review process.

Please submit your revised manuscript by Aug 19 2026 11:59PM. If you will need more time than this to complete your revisions, please reply to this message or contact the journal office at plosone@plos.org. When you're ready to submit your revision, log on to https://www.editorialmanager.com/pone/ and select the 'Submissions Needing Revision' folder to locate your manuscript file.

- A letter that responds to each point raised by the academic editor and reviewer(s). You should upload this letter as a separate file labeled 'Response to Reviewers'.
- A marked-up copy of your manuscript that highlights changes made to the original version. You should upload this as a separate file labeled 'Revised Manuscript with Track Changes'.
- An unmarked version of your revised paper without tracked changes. You should upload this as a separate file labeled 'Manuscript'.

If you would like to make changes to your financial disclosure, please include your updated statement in your cover letter. Guidelines for resubmitting your figure files are available below the reviewer comments at the end of this letter.

If applicable, we recommend that you deposit your laboratory protocols in protocols.io to enhance the reproducibility of your results. Protocols.io assigns your protocol its own identifier (DOI) so that it can be cited independently in the future. For instructions see: https://journals.plos.org/plosone/s/submission-guidelines#loc-laboratory-protocols. Additionally, PLOS ONE offers an option for publishing peer-reviewed Lab Protocol articles, which describe protocols hosted on protocols.io. Read more information on sharing protocols at https://plos.org/protocols?utm_medium=editorial-email&utm_source=authorletters&utm_campaign=protocols.

As the corresponding author, your ORCID iD is verified in the submission system and will appear in the published article. PLOS supports the use of ORCID, and we encourage all coauthors to register for an ORCID iD and use it as well. Please encourage your coauthors to verify their ORCID iD within the submission system before final acceptance, as unverified ORCID iDs will not appear in the published article. *Only*  the individual author can complete the verification step; PLOS staff *cannot*  verify ORCID iDs on behalf of authors.

We look forward to receiving your revised manuscript.

Kind regards,

Babak Ghazi, PhD

Academic Editor

PLOS One

Journal Requirements:

If the reviewer comments include a recommendation to cite specific previously published works, please review and evaluate these publications to determine whether they are relevant and should be cited. There is no requirement to cite these works unless the editor has indicated otherwise.

Please review your reference list to ensure that it is complete and correct. If you have cited papers that have been retracted, please include the rationale for doing so in the manuscript text, or remove these references and replace them with relevant current references. Any changes to the reference list should be mentioned in the rebuttal letter that accompanies your revised manuscript. If you need to cite a retracted article, indicate the article’s retracted status in the References list and also include a citation and full reference for the retraction notice.

Additional Editor Comments:

The overall quality of the manuscript has improved during the previous revisions. However, the reviewer believes that some modifications are still needed before the manuscript can be considered for publication. Therefore, the authors are requested to carefully revise the Abstract and Results sections to ensure that all statements are fully supported by the statistical analyses and are consistent with the reported regression results.

[Note: HTML markup is below. Please do not edit.]

Reviewers' comments:

Reviewer's Responses to Questions

**Comments to the Author**

Reviewer #1: (No Response)

**********

2. Is the manuscript technically sound, and do the data support the conclusions?

Reviewer #1: Partly

**********

3. Has the statistical analysis been performed appropriately and rigorously?

Reviewer #1: (No Response)

**********

4. Have the authors made all data underlying the findings in their manuscript fully available?

The PLOS Data policy

Reviewer #1: (No Response)

**********

5. Is the manuscript presented in an intelligible fashion and written in standard English?

Reviewer #1: Yes

**********

Reviewer #1: Thank you to the authors for revising the manuscript and responding to my previous comments. I appreciate the effort made to improve the paper. The manuscript has improved, particularly with the clarification of the abbreviation in the abstract and some revisions to the introduction. However, I believe a few important issues still need to be addressed before the manuscript is ready for publication.

Abstract: The additional information on age, gender, education, and marital status is not fully supported by the analysis presented. For example, statements such as "men dominate the adoption of CSA practices" and "married farmers are more likely to adopt" are based on the proportion of respondents in the sample rather than the regression results. Since gender was not statistically significant, these statements should either be removed or revised to reflect the actual findings. In addition, the statements about younger farmers adopting more are inconsistent with the regression results and should be checked carefully. Also, you used some references in the abstract, but I think using references in the abstract section is not allowed.

Consistency of results: There appear to be inconsistencies between the regression tables, the Results section, and the Abstract, particularly regarding the effect of age on CSA adoption. These interpretations should be checked carefully to ensure that they match the reported odds ratios.

Overall, the manuscript has improved, but I believe these issues should be addressed before the paper is accepted.

**********

what does this mean?). If published, this will include your full peer review and any attached files.

If you choose “no”, your identity will remain anonymous but your review may still be made public.

**Do you want your identity to be public for this peer review?**  For information about this choice, including consent withdrawal, please see our Privacy Policy

Reviewer #1: No

**********

[NOTE: If reviewer comments were submitted as an attachment file, they will be attached to this email and accessible via the submission site. Please log into your account, locate the manuscript record, and check for the action link "View Attachments". If this link does not appear, there are no attachment files.]

To ensure your figures meet our technical requirements, please review our figure guidelines: https://journals.plos.org/plosone/s/figures

You may also use PLOS’s free figure tool, NAAS, to help you prepare publication quality figures: https://journals.plos.org/plosone/s/figures#loc-tools-for-figure-preparation

NAAS will assess whether your figures meet our technical requirements by comparing each figure against our figure specifications.

# Author comment: Author response to Decision Letter 3

**DOI:** 10.1371/journal.pone.0355679.r006

22 Jul 2026

Response to Reviewers Comments

Challenges to Adoption of Climate-Smart Agricultural Practices Among Smallholder Farmers in Semi-Arid Ghana.

Abstract

Comment: The additional information on age, gender, education, and marital status is not fully supported by the analysis presented. For example, statements such as "men dominate the adoption of CSA practices" and "married farmers are more likely to adopt" are based on the proportion of respondents in the sample rather than the regression results. Since gender was not statistically significant, these statements should either be removed or revised to reflect the actual findings. In addition, the statements about younger farmers adopting more are inconsistent with the regression results and should be checked carefully. Also, you used some references in the abstract, but I think using references in the abstract section is not allowed.

Response: Thank you very much for this comment. We have revised the manuscript to include the regression results on the effect of age and education on CSA adoption. The section now reads;

Demographic factors also shaped the adoption of CSA practices. Farmers within the ages of 26-45 were less likely to adopt CSA practices (OR = 0.050; p<0. 05), while farmers with formal education were more likely to adopt CSA practices (OR = 9.262; p<0. 00) after controlling for motivational and support system variables.

Also, we have removed all the references in the abstract.

Consistency of results

Comment: There appear to be inconsistencies between the regression tables, the Results section, and the Abstract, particularly regarding the effect of age on CSA adoption. These interpretations should be checked carefully to ensure that they match the reported odds ratios.

Response: Inconsistent interpretations between regression tables, results section, and abstract have been resolved to match the reported odds ratios. The effect of age on CSA adoption is less likely. The result section now reads:

However, while no significant association was found between age and CSA in Model 1, in Model 2, farmers between the ages of 26-35 (OR=0.102; p<0.1) were less likely to adopt CSA practices.

Thank you very much for your constructive comments.

Attachment Submitted filename: Response_to_Reviewers_auresp_3.docx

# Editor report: Decision Letter 3

**DOI:** 10.1371/journal.pone.0355679.r007

**Authors:** Babak Ghazi

26 Jul 2026

Challenges to the Adoption of Climate-Smart Agricultural Practices Among Smallholder Farmers in Semi-Arid Ghana.

PONE-D-25-35144R3

Dear Dr. Molle,

We’re pleased to inform you that your manuscript has been judged scientifically suitable for publication and will be formally accepted for publication once it meets all outstanding technical requirements.

Within one week, you’ll receive an e-mail detailing the required amendments. When these have been addressed, you’ll receive a formal acceptance letter and your manuscript will be scheduled for publication.

An invoice will be generated when your article is formally accepted. Please note, if your institution has a publishing partnership with PLOS and your article meets the relevant criteria, all or part of your publication costs will be covered. Please make sure your user information is up-to-date by logging into Editorial Manager at Editorial Manager® and clicking the ‘Update My Information' link at the top of the page. For questions related to billing, please contact billing support.

If your institution or institutions have a press office, please notify them about your upcoming paper to help maximize its impact. If they’ll be preparing press materials, please inform our press team as soon as possible -- no later than 48 hours after receiving the formal acceptance. Your manuscript will remain under strict press embargo until 2 pm Eastern Time on the date of publication. For more information, please contact onepress@plos.org.

Kind regards,

Babak Ghazi, PhD

Academic Editor

PLOS One

Additional Editor Comments (optional):

Reviewers' comments:

# Editor report: Acceptance letter

**DOI:** 10.1371/journal.pone.0355679.r008

**Authors:** Babak Ghazi

PONE-D-25-35144R3

PLOS One

Dear Dr. Molle,

I'm pleased to inform you that your manuscript has been deemed suitable for publication in PLOS One. Congratulations! Your manuscript is now being handed over to our production team.

At this stage, our production department will prepare your paper for publication. This includes ensuring the following:

* All references, tables, and figures are properly cited

* All relevant supporting information is included in the manuscript submission,

* There are no issues that prevent the paper from being properly typeset

You will receive further instructions from the production team, including instructions on how to review your proof when it is ready. Please keep in mind that we are working through a large volume of accepted articles, so please give us a few days to review your paper and let you know the next and final steps.

Lastly, if your institution or institutions have a press office, please let them know about your upcoming paper now to help maximize its impact. If they'll be preparing press materials, please inform our press team within the next 48 hours. Your manuscript will remain under strict press embargo until 2 pm Eastern Time on the date of publication. For more information, please contact onepress@plos.org.

You will receive an invoice from PLOS for your publication fee after your manuscript has reached the completed accept phase. If you receive an email requesting payment before acceptance or for any other service, this may be a phishing scheme. Learn how to identify phishing emails and protect your accounts at https://explore.plos.org/phishing.

If we can help with anything else, please email us at customercare@plos.org.

Thank you for submitting your work to PLOS ONE and supporting open access.

Kind regards,

PLOS ONE Editorial Office Staff

on behalf of

Dr. Babak Ghazi

Academic Editor

PLOS One
